# The Impacts of Global Climate Change and Environmental Security on Fruits and Vegetables—A Policy–Technology Nexus Perspective

**DOI:** 10.3390/foods14234016

**Published:** 2025-11-23

**Authors:** Xuzeng Wang, Mengyang Xing, Jian Li, Boqiang Li

**Affiliations:** 1 Key Laboratory of Green and Low-Carbon Processing Technology for Plant-Based Food of China National Light Industry Council, Beijing Technology and Business University, Beijing 100048, China; 2 State Key Laboratory of Plant Diversity and Specialty Crops, Institute of Botany, Chinese Academy of Sciences, Beijing 100093, China; 3 China National Botanical Garden, Beijing 100093, China

**Keywords:** climate factors, fruit and vegetable industry, agricultural governance, global value chains, sustainable consumption and production

## Abstract

Global climate change exerts a systematic threat to the yield stability, nutritional quality, pest and disease control, and supply chain security of the fruit and vegetable industry via multiple ways, including altering temperature, carbon dioxide concentration, rainfall, ocean acidification, and soil deterioration. To tackle climate change, actions like carbon pricing and low-carbon technologies not only promote emission reduction but also impose pressure and economic difficulties on farmers, producers, logistics, transport, etc. This review, from an integrated view of “policy–technology relationship”, begins by summarizing the impacts of the aforementioned climate factors and systematically analyzes the influence of climate, policy, and technology on the fruit and vegetable industry. The research shows that the solution lies in the strategic cooperation between policies and technologies: technological innovation (e.g., controlled environment agriculture) offers potential for establishing resilient production systems, yet its successful implementation largely relies on forward—looking policy support and infrastructure investment, particularly the initial investment in renewable energy. Therefore, this paper puts forward an integrated framework intended for designing “resilient” fruit and vegetable systems, offering new theoretical foundations and path options for the coordinated advancement of climate mitigation and global nutrition security goals. This work offers an integrated framework for designing antifragile fruit and vegetable systems, harmonizing climate mitigation (SDG 13) with nutritional security (SDG 2) through strategically coordinated policy and technology interventions.

## 1. Introduction

Since the Industrial Revolution, the Earth has been undergoing continuous warming. During 2011–2020, the global average surface temperature was already 1.7 ± 0.1 °C higher than pre-industrial times (1700–1860) [1]. The change is mainly driven by anthropogenic greenhouse gas (GHG) emissions, especially in the form of carbon dioxide (CO_2_) [2]. Global climate change gives rise to serious negative changes to the atmosphere, hydrosphere, lithosphere, cryosphere, and biosphere, and causes more frequent occurrence of extreme weather events, sea-level rise, stronger abiotic and biotic stress to plants, etc. [3,4]. In response to global warming, a landmark United Nations climate agreement was signed in Paris in 2015. In the Paris Agreement, all the contracting countries agreed to limit warming below 1.5–2.0 °C by 2050 compared with the pre-industrialization level. Many countries have released roadmaps for GHG emission reduction. Some countries have announced reaching a carbon neutrality target by 2050, such as Japan, Brazil, the United Arab Emirates [5]. India has proposed the 2040 target of carbon neutrality and 2070 net zero [5]. Australia will reduce 26–28% of GHG emissions by 2030, compared to 2005 levels [6]. The European Union (EU) proposed the 2030 Climate Target Plan to reduce carbon emissions by 55% [7]. China plans to achieve carbon peaking by 2030 and carbon neutrality by 2060 [8]. Carbon pricing as a cost-effective emission reduction mechanism has also been implemented in many countries and regions.

Climate change brings huge challenges to food security. On one hand, the demand for food will continuously increase along with the growing world population, which is expected to reach 9.7 billion by 2050 and 10.9 billion by 2100. On the other hand, food production systems contribute about a third of anthropogenic GHG emissions, and more than half of GHG emissions are from animal-derived food production [9,10]. During the past 20 years, the GHG emissions from agricultural food production systems still increased by around one-third due to the increased livestock and crop production [11]. The development of sustainable agricultural technologies is required to resolve this contradiction. In addition, changing diet habits to less livestock-based but more plant-based foods is also considered an effective way to decrease GHG emissions and mitigate global warming.

The climate is one of the primary driving factors for the growth and distribution of living organisms [12]. Plants exhibit high sensitivity to climate change [13]. The fluctuations in climatic factors, mainly including temperature, atmosphere, and precipitation, impact various aspects of plants, such as phenological stages, physiological processes, metabolic activities, pest and disease occurrence, yield, and the qualitative composition of plant tissues and derived products [14,15].

Fruits and vegetables are crucial components of plant-based foods. The World Health Organization (WHO) recommends an intake of 400 g/day of fruits and vegetable products [16]. Fruits and vegetables are generally low in energy density and rich sources of essential nutrients include antioxidants, vitamins (especially vitamins C and A), minerals, and fiber [17]. Sufficient amounts of fruit and vegetable consumption play a vital role in human health by potentially preventing diseases such as cardiovascular diseases (CVDs), obesity, and type 2 diabetes mellitus [18]. Low intakes of fruits and vegetables are the third and fifth main factors leading to diet-related deaths, mainly caused by cardiovascular diseases, type 2 diabetes, and neoplasms worldwide. To achieve a sustainable and healthy diet for 10 billion people, global fruit and vegetable production will need to increase by 50–150% in 2050 [19]. Compared to animal husbandry and other crop production, the carbon emissions of the fruit and vegetable industry are lower. The mean carbon footprint (CF) of plant-based foods is only 10.7% of that of animal-based foods [20]. Beef has the highest CF of 15.0 kg CO_2_-eq/kg in animal-based foods. In crop production, rice has the highest CF of 1.31 kg CO_2_-eq/kg in plant-based foods, while the mean CFs of vegetables and fruits are only 0.57 and 0.39 kg CO_2_-eq/kg, respectively. However, in the context of global carbon emission reduction, emission reduction measures still need to be applied to the production of fruits and vegetables and the supply chain after harvest.

In this review, the impacts of temperature, CO_2_ concentration, water, and soil pH caused by global climate change on fruit and vegetable yield, quality, disease and pest control, and food safety are summarized. The impacts of carbon pricing policies on the industry are discussed. Finally, the main countermeasures and available technologies for low-carbon development of the fruit and vegetable industry are suggested.

## 2. Methodology

To ensure a comprehensive, transparent, and reproducible analysis of the existing literature on the impacts of global climate change and carbon pricing policies on the fruit and vegetable industry, this review was conducted following a systematic approach. While it is a narrative synthesis that aims to cover a broad and complex policy–technology nexus, rather than a meta-analysis targeting a single quantitative effect size, rigorous steps were undertaken to minimize bias and enhance the validity of the findings.

### 2.1. Literature Search and Source Identification

A systematic literature search was performed to identify relevant peer-reviewed publications. Primary searches were conducted using major academic databases, including Web of Science Core Collection, Scopus, and Google Scholar. The search strategy combined keywords and Boolean operators related to three primary themes: (1) climate factors, (2) fruit and vegetable systems, and (3) impacts and policies. Key search strings included: (“climate change” or “global warming” or “temperature increase” or “CO_2_ enrichment” or “precipitation change” or “extreme weather”) and (“fruit” or “vegetable” or “horticultur”) and (“yield” or “quality” or “pest” or “disease” or “food safety” or “carbon footprint”). Additionally, specific searches were conducted for policy mechanisms using terms such as (“carbon pricing” or “carbon tax” or “emissions trading” or “ETS”) and (“agricultur” or “food supply chain”). The search was limited to articles published in English and Chinese between January 2000 and March 2024 to capture the most relevant and contemporary research.

### 2.2. Study Selection and Screening Criteria

The initial pool of search results was screened in a two-stage process based on pre-defined inclusion and exclusion criteria. The inclusion criteria required that studies (i) empirically investigated or modeled the impact of at least one defined climate variable (e.g., temperature, CO_2_, water stress, soil salinity) on the production, quality, safety, or economic aspects of a fruit or vegetable crop; or (ii) analyzed the effects, implications, or case studies of carbon pricing policies within the agricultural or food supply chain, with a preference for studies focusing on the fruit and vegetable sector. Review articles were consulted for foundational knowledge and to identify additional primary sources, but were not the primary basis for impact conclusions.

Studies were excluded if they (i) focused solely on staple grains or animal husbandry without relevant parallels to horticulture; (ii) were purely opinion pieces without empirical data or robust modeling; or (iii) were not accessible in full text. Titles and abstracts were screened first, followed by a full-text review of the remaining articles to assess their eligibility based on the criteria. This process, summarized conceptually, aimed to ensure the selected literature was directly pertinent to the review’s objectives.

### 2.3. Data Extraction and Synthesis

Key information was extracted from the included studies into a structured framework. This included: the climatic factor or policy instrument examined, the specific fruit or vegetable crop, the geographical context of the study, the main methodological approach (e.g., field experiment, controlled environment study, economic model, LCA), the primary findings regarding impact (on yield, quality, etc.), and the core conclusions. Given the heterogeneous nature of the studies, spanning agronomy, food science, economics, and policy analysis, a narrative synthesis approach was adopted. This involved identifying, categorizing, and summarizing the main themes, patterns, and consensus or disagreement within the literature. The synthesis aimed to provide a coherent overview of the complex interactions within the system rather than a statistical aggregation.

### 2.4. Acknowledgment of Scope and Limitations

This methodology inherently shapes the scope and conclusions of the review. The reliance on published academic literature means that the review reflects the geographic and thematic biases present in the research landscape. There is a heavier representation of studies from temperate and semi-arid regions (e.g., North America, Europe, parts of Asia) compared to tropical, Mediterranean, or mountainous agro-ecological zones. Furthermore, the available literature is more concentrated on the effects of temperature, CO_2_, and water, with comparatively fewer empirical studies on the isolated and combined impacts of UV-B radiation, tropospheric ozone, and soil pH/salinity on quality and safety parameters. This imbalance in the literature base, rather than an oversight in the review process, explains the differential depth of coverage across climate factors in the subsequent sections. The review explicitly acknowledges these gaps as critical areas for future research in the conclusion. This systematic and transparent methodology provides a foundational rationale for the structure of the evidence presented and the ensuing discussion on policy and technology.

## 3. Impacts of Global Climate Change on Fruit and Vegetable Industry

### 3.1. Impacts of Global Climate Change on Fruit and Vegetable Yield

Crop yield is of great significance to meeting people’s dietary requirements. The yield of fruits and vegetables is heavily affected by climate factors, including temperature, CO_2_ concentration, rainfall, and soil pH.

#### 3.1.1. Temperature

**Figure 1:**
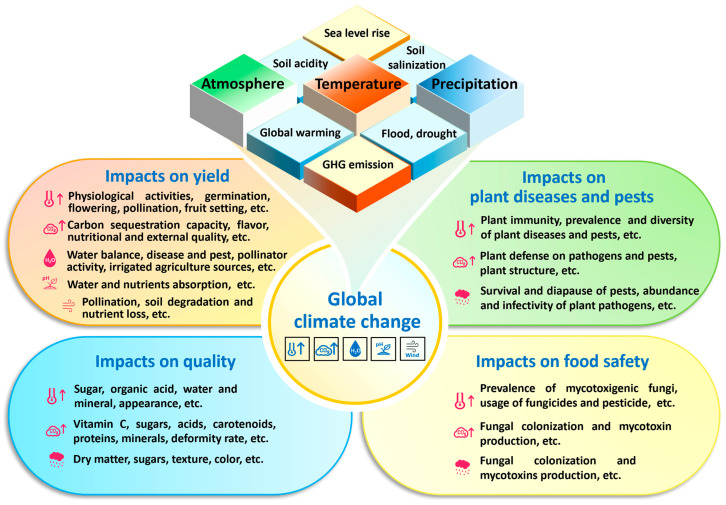
The impacts of global climate change on fruit and vegetable supply.

The continuous global warming and frequent extreme climate events have a serious impact on fruit and vegetable planting and harvest (Figure 1). Several studies have demonstrated that environmental temperature is the most important factor affecting the growth and harvesting process of fruits and vegetables [21,22]. High temperatures can affect photosynthesis, respiration, water stress, and membrane stability of crops, as well as levels of plant hormones, primary and secondary metabolites, and even inhibit seed germination under extreme heat conditions. Haque et al. [23] found that high temperatures (≥36 °C) had dominant effects on the yield of the three kinds of fruits (pineapple, mango, and lychee) in certain areas of Central Queensland. Among them, mango was the most sensitive to environmental conditions. In addition, pineapple yield decreased by about 6% for every 1 °C-temperature change (Table 1). High temperatures also reduce the fruit setting rate, single fruit weight, and fruit number per plant of tomato, and may cause a yield reduction by 70% [24].

In the whole process of plant growth, the temperature before flowering is more important than that before harvest. The 46-year (1976–2021) large-scale data from the Wakayama prefecture in Japan was used to predict Japanese apricot yield using the state-space model and a time series model of fruit yield using 15 years of fruit setting data (2007–2021) [25]. The results showed that the temperature before flowering had significant effects on the fruiting rate and fruit yield. If the average temperature increased by 1 °C, the fruit yield would decrease by 100 kg/1000 m^2^ on average. In addition, the authors argued that cultivation management interventions on fruit set rate and fruit growth should also be considered. The influence of phenology, suitability, and distribution of peach was studied in the whole French mainland [26]; it reached a consistent conclusion: an increase in temperature during the flowering season will reduce fruit yield.

Abnormal temperature fluctuation caused by global climate change also affects the phenological characteristics of fruits and vegetables. Several peach varieties’ growth and yield data were obtained for five years in the Monag region of northern Tunisia (36°41′ N, 10°15′ E) [27]. The abnormal temperature rise in winter affected the flowering of peaches, resulting in bud shedding, reduced fruit setting, and decreased fruit yield.

#### 3.1.2. CO_2_

Plants convert CO_2_ into glucose through photosynthesis, providing organic matter for plants. Therefore, the concentration of CO_2_ in the atmosphere has a great impact on crop yield. CO_2_ concentration in the atmosphere affects the yield of fruits and vegetables from many aspects, including the primary photochemistry, photoprotection mechanism, and carbon sequestration capacity of plants [38]. A study [28] about the plant response parameters of tomatoes under different CO_2_ concentrations indicated that 700 ppm CO_2_ had the greatest stimulating effect on the transfer of photosynthetic products to fruit. Under reduced irrigation conditions, elevated CO_2_ (800 ppm) could improve the development and nitrogen uptake efficiency of cherry tomato roots, increase irrigation water productivity, and thus increase yield [29].

#### 3.1.3. Water

Precipitation is expected to increase by about 7% for every 1 °C of warming. A warmer atmosphere can hold more moisture, so a warmer climate may produce more frequent rain and snow. The change in rainfall has significant effects on plant physiology and pollination activities of fruits and vegetables.

The growth of dragon fruit cultivated on the Deccan Plateau of peninsular India was evaluated in the past eight years [30]. They found that higher rainfall during bud formation and flowering could cause the drop of dragon fruit buds or flowers and fruit decay, thereby reducing fruit production. Moreover, continuous rainfall led to lower soil oxygen levels, and higher humidity caused fruit fly decay, which further reduced fruit production. The effects of rainfall and harvest time on triploid red Pomelo × grapefruit hybrid (Redson) in 2022–2023 were studied; the results showed that various rainfall parameters were strongly correlated with harvest time and rot incidence. Overall, early-harvested fruits had a superior postharvest storage performance in the fall, while late-harvested fruits suffered from the development of decay during the rainy winter months [39]. Since wind plays little or no role in the pollination process of loquat, it is mainly pollinated by bees and other insects. Frequent rainfall will reduce or inhibit pollinator activity, reduce the fruit setting rate of loquat, and ultimately lead to the decline of loquat yield. Flooding and waterlogging caused by extreme climate change events have a more serious impact on the yield of fruits and vegetables. When the soil is saturated with excess water, the diffusion of gas through the soil is reduced, which reduces O_2_ supply to the roots and causes damage to the plant root system. Flooding resulted in reduced peach branch and fruit growth was reported [40], accompanied by multiple plant physiological dysfunctions.

Climate change can affect water levels of the rivers and lakes, which provide important agricultural irrigation sources. In recent decades, many lakes in the Tibet Plateau have rapidly expanded due to the melting of mountain glaciers caused by global warming, whereas some lakes in arid areas have shrunk or even vanished [41]. Climate change affects the transition of flood and dry seasons in Poyang Lake, the largest freshwater lake in China [41]. Irrigated agriculture may suffer negatively from lack of dry-season water, and it may lead to yield losses.

#### 3.1.4. Soil pH and Salinity

Climate change causes alterations in soil pH. In general, alkaline soils are common in arid climates, while acidic soils are common in humid climates [42]. Fruit and vegetable crops growing in excessively acidified and salinized soils are affected by osmotic stress, nutritional disorders, and toxicity [43], which will seriously reduce crop yield.

Soil salinity levels determine crop yield levels. At very low salinity levels, fruit and vegetable yields are not affected, while medium and high salinity levels (≥4 dS/m) will reduce the osmotic potential of soil water and limit the absorption of water and nutrients by plant roots, resulting in physiological drought and a decrease in fruit and vegetable yield, that is, osmotic stress of soil salinity [44]. Long-term experiments on 18 local and regional date palm varieties were conducted in the Arabian Peninsula [32]. The results showed that the yields of all date palm varieties were significantly negatively affected under 3 salinity levels (5, 10, and 15 ds/m). Among them, the average yield of all varieties under the 10 ds/m condition was reduced by 44%, the average yield of the 15 ds m^−1^ condition was reduced by 64%, and the maximum reduction ratio reached 67%. For the Mediterranean coastal regions, soil secondary salinization is a major characteristic. Research [45] on the important vegetable pepper in Spain indicates that salt stress (5.8 dS m^−1)^ has a more severe inhibitory effect on yield than the same level of water stress. However, by using germplasm from arid regions (such as ‘Numex Heritage’) or certain wild relatives of peppers (such as C. baccatum U27610) as rootstocks, the grafted plants can maintain higher fruit weight and yield under salt stress.

Overall, many field studies conducted over the past decade in a variety of geographical and climatic conditions around the world have found that soil pH can lead to a decrease in crop yields, with a linear change in crop yield with an increase in soil acidification or alkalization.

#### 3.1.5. Wind

Climate change will impact the geographic distribution of wind, change wind speed, and may bring more frequent hurricane landfalls in certain locations. The wind has long been recognized as an important factor influencing plant transpiration, pollination, and seed spread. During the flowering phase, high-speed wind can result in flower drop and pollination failure by coupling with other climate factors. A study found that hurricanes and other factors were devastating to Florida’s fruit industry [46], with total citrus production dropping from 291.8 million boxes in 2003–2004 to 49.58 million boxes in 2017–2018, acreage dropping to 400 and 900 acres, and the value of citrus on trees dropping 47% ($513 million). The decline in Florida grapefruit production was even greater, down 90% (1.573 million tons) over the same period, with 52,500 fewer acres planted. The authors suggest that hurricane damage to barriers and other facilities, leading to an increase in the number of *Diaphorina citri* in citrus trees, resulting in huanglongbing (HLB) disease, is an important cause. Another study found that an unusually wet season (21 cm of rain in 11 h), affected by Hurricane Wilma in 2017 [47], resulted in high soil nitrogen loss and soil salt loss (electrical conductivity > 2500 μS cm^−1^) in Florida; as a result, local crop yields have fallen. Due to the extensive crop damage (wind and flooding) caused by hurricane Wilma, the national market price for tomatoes reached its highest in the following fall.

#### 3.1.6. UV Radiation

While often less discussed than temperature or CO_2_, ultraviolet (UV) radiation, particularly UV-B (280–315 nm), is a significant factor modulated by climate change and stratospheric ozone depletion [48]. Elevated UV-B radiation can directly impact the yield of fruits and vegetables by impairing fundamental physiological processes. Compared to the control group (24 kJ/(m (2) center dot d)) [49], fruit yield, soluble sugar, sugar-acid ratio and vitamin C of ‘Tainong No. 1’ mango (Mangifera indica) trees all decreased under the 96 kJ/(m (2) center dot d) treatment, while no significant changes were observed in the control group. After 20 days or 40 days, the net photosynthetic rate (Pn), stomatal conductance (Sc), transpiration rate (Tr), intercellular CO_2_ concentration (Ci) and chlorophyll a/b of the leaves exposed to 96 kJ/(m (2) center dot d) UV-B radiation were significantly lower than those of the control group. The author believes that 96 kJ/(m (2) center dot d) directly led to stomatal limitation, resulting in inhibition of photosynthesis, reduced yield, and deterioration of mango quality.

Some scholars have also discovered through research that the combined effect of multiple types of light can enhance the antioxidant and other characteristics of crops. By using the mathematical modeling method, it was discovered [50] that the sole UV-B radiation (peak at 309 nm; luminous flux of 5 μmol m^−2^ s^−1^) promoted tomato yield, as well as the increase in ascorbic acid (AsA) and most hydroxycinnamic acids (HCAs). The combined supplementation of blue light B (peak at 444 nm; luminous flux of 238 μmol m^−2^ s^−1^) + UV-B light treatment made the fruits harder and had a high HCA content, and the accumulation of rapid-acting antioxidants significantly increased. Sun et al. [51] also discovered similar results. By using a combined treatment of UV-B radiation and water deficit, they significantly increased the amino acid content in the grape Vitis vinifera L. cv. Pinot Noir, especially proline (Pro), aspartic acid (Arg), alanine (Ala), and threonine (Thr).

As climate change continues to influence atmospheric dynamics, understanding the yield implications of fluctuating UV-B levels becomes crucial for breeding resilient varieties and managing horticultural production.

#### 3.1.7. Ozone

Ground-level ozone (O_3_) is a potent phytotoxic air pollutant whose concentrations are rising in many regions due to increased emissions of precursor compounds (e.g., nitrogen oxides and volatile organic compounds) under warmer climatic conditions [52]. Unlike the protective stratospheric ozone, tropospheric O_3_ negatively impacts plant physiology and productivity. Hui et al. [53] conducted a long-term study on the wine grape ‘Cabernet Sauvignon’ (with 75 ppb O_3_ for three months) and discovered that O_3_ stress not only reduced the plant biomass and fruit quality, but also severely damaged the photosynthetic system, especially decrease in the actual photochemical efficiency of PSII and PSI, and the total absorption of C-13 and N-15 by the leaves. Another experiment also yielded similar results [54]. Tomato plants with 30-day and 51-day seedling ages were exposed to high concentrations of O_3_ (100–250 ppb), visible leaf damage, inhibited internode growth, and decreased total root length and number were all observed. O_3_ alters the distribution of dry matter, inhibits root development, and delays growth, ultimately leading to a reduction in tomato yield. Moreover, the degree of damage and recovery of the crops highly depends on the developmental stage when they are subjected to stress.

#### 3.1.8. Ocean Acidification

Ocean acidification (OA) is mainly caused by the absorption of anthropogenic CO_2_ by the ocean, resulting in a decrease in the pH value of seawater and directly affecting the growth and survival of calcifying organisms (such as corals, shellfish, etc.) in the ocean [55]. In addition, OA may also indirectly affect some coastal agriculture that relies on marine resources by altering the environment of the near-shore areas. On one hand [56], acidification changes the forms and bioavailability of trace elements in seawater, and through processes such as sea fog and atmospheric deposition, these elements can enter the coastal agricultural system, potentially affecting the soil nutrient status. On the other hand [57], ocean acidification may also interfere with the nutritional components and physiological functions of seaweed used as fertilizer, thereby affecting its application effect in agriculture. Although there is currently insufficient literature and direct evidence to indicate that ocean acidification has significantly threatened the land-based fruit and vegetable industry, its potential indirect impacts on marine ecology and coastal agricultural systems still warrant continuous attention and research.

### 3.2. Impacts of Global Climate Change on Fruit and Vegetable Quality

Global climate change not only affects the production of fruits and vegetables but also affects their quality, thereby affecting the nutritional intake of humans.

#### 3.2.1. Temperature

Temperature affects the content of various components of fruits and vegetables, which will change the basic properties such as taste, flavor, nutrition, and skin color, and affect the commercial or edible value. Elevated temperature decreased the soluble solids content in apple fruit, but increased titratable acidity [33]. Fruit skin color was abnormal under high temperatures. In addition to sugar and organic acid content, water and mineral content are also affected by rising ambient temperatures. High-temperature growing environments (+3 °C) increased the content of some major minerals (K, P, S, Ca, and Fe) in tomato (*Solanum lycopersicum* cv. ChapingoF1) [58], but reduced P, S, and Ca content in cultivar ‘Money Maker’. The antioxidants in fruits and vegetables also change when exposed to high temperatures during the growing season. Strawberries grown at 30/20 °C (day/night) had significantly higher antioxidant capacity [35], compared to the fruits grown at 25/20 °C, and total polyphenols content increased by about 80%.

Temperatures higher than normal will cause harm to fruits and vegetables, affecting their appearance and edible quality. High temperatures (36 °C/28 °C, day/night) [59] inhibited the growth of kumquat fruit, resulting in reduced fruit weight and size. Moreover, the color of the fruit peel could not change from green to orange. Now, the frequency of extremely high temperatures is increasing, and the affected areas are becoming wider and wider. Due to global warming, the impact of high-temperature stress on the quality of fruits and vegetables will become more severe.

The impact of high temperatures on the quality of fruits and vegetables is particularly significant in tropical and semi-arid regions. Taking the South African cactus pear as an example [60], nopalitos harvested in the spring under higher temperatures (average maximum 30.4 °C exhibit superior texture and flavor: they have a higher penetration value (more tender), lower titratable acidity (0.35% vs. 0.39% in autumn), and a brighter green color (higher L* and C values). In contrast, samples harvested in autumn, due to lower temperatures, have a harder texture and higher acidity. The Morado variety shows stronger temperature adaptability in spring, with lower mucilage viscosity, reducing the unpleasant stickiness. This indicates that in a warming climate, selecting heat-tolerant varieties and optimizing the harvest season can effectively maintain the sensory quality of fruits and vegetables, especially for specialty crops in arid areas.

#### 3.2.2. CO_2_

Atmospheric CO_2_ concentration will change the net photosynthesis, stomatal conductance, hardness, nutrient use efficiency, and plant water potential. Moderately increasing the CO_2_ concentration can improve the photosynthetic efficiency and quality. The increase in CO_2_ concentration possibly improves the content of vitamin C, sugars, organic acids, and pigments, and has a positive impact on the total antioxidant capacity, phenols, and anthocyanins in various fruits and vegetables [61]. Elevated CO_2_ concentration improved the contents of soluble solids, vitamin C, and lycopene in tomato fruit, which may be caused by increased photosynthesis under high CO_2_ levels [62].

However, high levels of CO_2_ also have negative effects on the quality. The experiment was defined as a randomized split-plot design with CO_2_ as the main plot and N supply as the subplot. The CO_2_ concentrations were targeted at 400 (ambient CO_2_), 800 (elevated CO_2_, eCO_2_), and 1200 µmol mol^−1^ (super-elevated CO_2_) in each chamber. To minimize the potential environmental variations among chambers, plants were randomly rotated within a 1-week interval [63]. Compared with the concentration of CO_2_ in the control group, the high concentration of CO_2_ generally increased the accumulation of carbohydrates in fruits, but the contents of minerals, nitrates, and proteins did not further increase. The increase in CO_2_ concentration may reduce the contents of proteins and minerals in products. Another experiment was conducted at the Department of Science at the University of Copenhagen, Taastrup, Denmark. The tomato seeds were planted on 14 December 2017, with the average of CO_2_ concentration, temperature (T), relative humidity (RH), and vapor pressure deficiency (PVD) designated at 400 and 800 ppm during the experimental period [64]. When CO_2_ concentration increased from 380 ppm to 550 ppm, the deformity rate of potato tubers increased by about 63%, resulting in poor processing quality. Moreover, higher CO_2_ concentration increased the probability of common scab by 134%.

#### 3.2.3. Rainfall

Abnormal rainfall caused by climate change has a severe negative impact on the quality of fruits and vegetables. A large amount of rainfall will reduce the original dry matter and texture of fruits. Excessive rainfall during fruit development increased the water content of guava in the Andes, thus making the fruit softer in texture and lower in sugar content [37]. One reason is that the long-term rainy weather may reduce sunlight exposure, resulting in a reduction in photosynthetic products (sugar, cellulose, etc.). High relative humidity prevents full transpiration of the fruit tree, resulting in poor absorption of nutrients absorbed by the mass flow. Rainfall is also a key environmental factor influencing the grape ripening process in the Mediterranean mountainous regions. In the Valtellina area [65], the cumulative precipitation throughout the growing season is positively correlated with the ripening time. For every 100 mm increase in precipitation, the date of reaching technical maturity is postponed by approximately 2 days, and the total acidity increases by about 1 g/L. Especially during the period when the fruits are expanding and ripening, the increase in precipitation lengthens the ripening period and raises the fruit acidity, which may be related to the sugar dilution and acid accumulation caused by sufficient water supply. This result suggests that in the management of mountain vineyards, water regulation has significant practical significance for balancing sugar-acid metabolism and optimizing the harvest time.

#### 3.2.4. pH and Salinity

Soil pH and salinity are critical determinants of fruit and vegetable quality, influencing nutrient availability, osmotic balance, and metabolic activity. Excessive salinity (EC ≥ 4 dS/m) can lead to osmotic stress and specific ion toxicity, impairing the accumulation of sugars, organic acids, and pigments. For instance, in tomato, soil salinity above 6 dS/m significantly reduces lycopene and β-carotene content, thereby diminishing nutritional value and visual appeal [43]. Similarly, strawberry plants grown under saline conditions (8 dS/m) exhibit a 15–20% decrease in soluble solids and anthocyanin content, adversely affecting flavor and antioxidant capacity [66]. In date palm, salinity stress (10–15 dS/m) not only reduces yield but also leads to poorer fruit texture and lower sugar content, impacting marketability [32]. Acidic soils (pH < 5.5) can induce aluminum and manganese toxicity, leading to chlorosis and reduced vitamin C in crops like bell pepper and cabbage [42]. Moreover, soil alkalinity (pH > 8.0) limits iron and phosphorus availability, causing leaf yellowing and poor fruit development in grapes and citrus [15]. These alterations in mineral uptake directly affect the sensory and nutritional quality, underscoring the need for soil amendment strategies such as gypsum application or organic matter incorporation to mitigate adverse effects.

#### 3.2.5. Wind

Wind, particularly under climate change-induced extreme weather events, exerts both physical and physiological impacts on fruit and vegetable quality. Strong winds cause abrasion and micro-injuries on fruit surfaces, facilitating water loss and pathogen entry. For example, in apple orchards, persistent wind at speeds >5 m/s leads to russeting and cuticle damage, reducing grade quality and postharvest longevity [67]. In grapevines, wind stress during berry development can reduce skin thickness and anthocyanin accumulation, compromising wine color and quality [65]. Wind also accelerates evapotranspiration, leading to water stress even in irrigated systems, which can alter fruit composition. Studies on citrus have shown that wind-exposed fruits have lower juice content and higher titratable acidity due to moisture deficit [46]. In leafy vegetables like lettuce, wind-induced mechanical stress triggers lignification, resulting in tougher texture and reduced palatability [14]. Furthermore, wind-borne salt spray in coastal areas can cause tip burn and necrosis in crops like tomato and strawberry, impairing both yield and visual quality [68]. These effects highlight the importance of windbreaks and sheltered cultivation systems to preserve product integrity.

#### 3.2.6. UV Radiation

UV radiation, especially UV-B, has been proven to improve the quality features of fruits and vegetables. It does so by facilitating the accumulation of health—beneficial compounds and enhancing sensory traits. In pepper fruits, exposure to UV-B triggered anthocyanin biosynthesis, causing the peel to turn purple. This process was controlled by transcription factors like CaMYB113, CabHLH143, and CaHY5, which directly attach to anthocyanin structural gene promoters, boosting anthocyanin content and related gene expression [69]. Likewise, in Pinot Noir grapes, UV-B exposure elevated skin anthocyanins and total phenolic content without notably changing sugar levels or total amino acids, implying a specific upregulation of flavonoid pathways [70]. In strawberries, pre-harvest UV-B treatment enhanced fruit color, raised total soluble solids (TSS), total phenolic content (TPC), and total anthocyanin content (TAC), and preserved firmness during storage. Gene expression analysis revealed that UV-B upregulated crucial anthocyanin biosynthesis genes such as FaMYB10, FaCHS1, FaF3H1, and FaUFGT at the red stage, while FaHY5 was more sensitive in earlier developmental phases [71].

Excessive UV-B radiation also has a negative impact on the quality of fruits and vegetables. The most direct manifestation is the occurrence of photostress and photoxidative damage, which leads to the appearance of burn marks, color fading or accelerated aging on the fruit surface, seriously affecting their commercial value. For example [71], although low-dose UV-B is beneficial, high-intensity or prolonged UV radiation may inhibit photosynthesis, affect the normal development and sugar accumulation of fruits, resulting in reduced yield or abnormal fruit shapes.

#### 3.2.7. Ozone

Increased ground-level ozone (O_3_) has complex and often contrasting effects on the quality of fruits and vegetables. On the positive side, several studies indicate that controlled ozone exposure can enhance certain bioactive compounds in produce. For instance [72], ozonation of radish plants before harvest was found to significantly increase the content of ascorbic acid, total polyphenols, and antioxidant activity during storage, particularly at doses of 1–5 ppm for short durations. This suggests that ozone can act as an elicitor, stimulating defense mechanisms that lead to the accumulation of health-promoting phytochemicals. Similarly, in tomato plants, ozone exposure altered dry matter partitioning and root-shoot ratios, which may indirectly influence nutritional composition [73]. On the negative side, however, elevated ozone concentrations often impair overall plant growth and yield quality. For example [74], cherry radish exposed to elevated ozone (70–140 ppb above ambient) showed a significant reduction in total biomass and hypocotyl size, the edible part by up to 49%, along with a shift in non-structural carbohydrate allocation away from storage organs toward leaves. This reallocation can reduce the economic and nutritional value of root vegetables. In tomatoes, ozone stress also led to shortened internodes and delayed growth, which may affect both visual and market quality [54]. Thus, while ozone can enhance certain phytochemical traits under controlled conditions, its overall impact on crop quality is often negative, particularly when exposure occurs during sensitive developmental stages.

#### 3.2.8. Ocean Acidification

Currently, there is a scarcity of literature directly studying the impact of OA on the quality of fruits and vegetables. However, we can infer this from the extensive influence of OA on the global ecosystem. OA works in synergy with climate change, intensifying global warming and leading to more frequent heat waves, droughts, and extreme precipitation events [75]. These environmental stresses directly affect the physiological processes of fruits and vegetables. For instance, high-temperature stress promotes the respiration of fruits [76], accelerates ripening and softening, resulting in a shorter storage period and shorter shelf life. Water stress affects fruit expansion, causing fruits to become smaller, irregular in shape, and potentially reducing sugar, vitamin, and antioxidant accumulation, thereby affecting flavor and nutritional quality [77]. Secondly, the chemical changes caused by OA in the ocean affect the transport of trace nutrients from the ocean to the land via sea salt aerosols. The ocean is an important source of certain trace elements, and changes in their chemical forms may affect the biological availability of these elements in coastal soil [78], thereby influencing the absorption of trace elements such as zinc and selenium by fruits and vegetables, and ultimately altering the nutritional value of the products. Therefore, although there is no direct exposure to acidified seawater, the quality formation process of coastal fruit and vegetable production areas is likely to be challenged by the climate and biogeochemical cycle changes driven by OA.

### 3.3. Impacts of Global Climate Change on Diseases and Pests of Fruits and Vegetables

Plant diseases and insect pests cause significant losses to agricultural production worldwide. The effects of climate change on plant diseases and insect pests are complex, as multiple aspects of host plants, pathogenic microbes/insect pests, and the environment are involved. Meteorological factors, such as temperature, CO_2_ levels, and moisture, may have a significant impact on promoting or inhibiting the occurrence, development, prevalence, and degree of damage of certain diseases and pests.

#### 3.3.1. Temperature

As high-temperature stress intensifies, plants exhibit adverse symptoms such as wilting, leaf burning, folding, and shedding. Additionally, physiological reactions such as RNA metabolism, protein synthesis, enzyme activity, and plant growth hormones also change. These alterations of host plants can suppress their immunity, thereby increasing the risk of pathogen and pest infection [79].

For pathogens, as temperatures rise, the growing seasons become longer, allowing for more time for pathogen evolution. Therefore, an increased likelihood of pathogens occurred during both winter and summer [80]. According to a recent investigation, it has been predicted that the occurrence of fungal pathogens such as *Alternaria*, *Fusarium*, *Penicillium*, and *Phytophthora* is expected to rise due to global warming [34]. Other examples of the impact of high temperatures on disease incidence include potato late blight, canola stem blight, and grape berries gray mold [80,81]. In these cases, high temperatures, along with high humidity, exacerbate disease severity. Furthermore, spore germination of *Penicillium* spp. increases with increasing temperature over a certain temperature range [82], and the fungal species *Monosporascus cannonballus* and *M.eutypoides* reproduce faster as the temperature rises, which have been described as the causal agents of *Monosporascus* root rot and vine decline disease (MRRVD), and mainly affects melon and watermelon crops [83].

In terms of insect pests, rising temperatures may lead to the emergence of new pests as well as pest niches. The growth and progression of insects are contingent on the temperatures they encounter. Since they are unable to regulate their body temperature, any temperature variation will directly affect the insect’s development [84]. On the other hand, temperature can significantly affect the insect’s egg stage, life span, and fertility of insects. A study based on *Scopula subpunctaria*, a deciduous pest of tea tree in China [85], showed that the average life span of female adult insects increased from 10.8 days at 33 °C to 33.9 days at 13 °C. At 33 °C, the number of female eggs was 15.4 compared to 279.9 eggs at 22 °C. Furthermore, high temperatures also indirectly affect the occurrence of pests and diseases by altering the physiological state of plants. In the Campania olive groves [86], high temperatures cause changes in the behavior of leaf stomata and alterations in the epidermal wax structure, which may increase the risk of pathogen invasion. Although the incidence of pests and diseases was not directly studied, the metabolic changes induced by high temperatures in plants (such as the accumulation of polyphenols) may affect their resistance to pests and diseases. This response is particularly evident in Mediterranean olive varieties, suggesting that in regions with frequent high temperatures, a combination of variety resistance and cultivation management should be adopted to address the risk of pests and diseases in the future.

In addition, global warming will expand the geographical distribution of pathogens and pests, which exposes them to additional potential plant hosts and offers new opportunities to hybridize [68,86]. An increasing amount of evidence indicates that newly invaded areas and new hosts suffer greater damage from pathogens and pests compared to their original areas and hosts [80].

#### 3.3.2. CO_2_

There is remarkable variability in the impacts of elevated CO_2_ levels on pathogen infection rates and the overall severity of diseases. Elevated CO_2_ levels intensify the severity of powdery mildew on cucurbits caused by *Sphaerotheca fuliginea*, but reduce the susceptibility of soybeans to the downy mildew pathogen *Peronospora manshurica* [87,88]. While higher CO_2_ levels generally increase absolute plant growth, they also reduce plant nutrition and alter plant physiology, resulting in less efficient plant defense pathways [89,90].

Impacts of elevated CO_2_ on the nutrient quality of plants will undoubtedly lead to higher plant consumption rates in certain pest groups, resulting in increased levels of plant damage as pests need to consume more plant tissue for an equivalent level of food [91]. *Spodoptera litura* is commonly found on beets, cruciferous and nightshade vegetables, causing damage by larvae feeding on the leaves, and has been reported emerging as a serious pest under higher levels of CO_2_ [84]. The impact of elevated CO_2_ concentration on crop diseases and pests is unique in arid-zone crops. The growth data of cactus pear in the semi-arid environment of South Africa [60] shows that its cladodes have a lower incidence of diseases under natural CO_2_ conditions (≈400 ppm), which may be related to the thickness of the epidermis and the physical barrier effect of mucilage. Although high CO_2_ was not directly tested, related studies have pointed out that the photosynthetic efficiency of cactus pear can still be maintained under drought stress, indirectly reducing its sensitivity to diseases and pests. Moreover, its mucilage component (if it is a glycoprotein) may inhibit the germination of pathogen spores. This suggests that in the context of rising CO_2_ levels, the inherent resistance of drought-tolerant crops may provide a natural strategy for disease and pest management, especially suitable for tropical and semi-arid agricultural systems with limited resources.

#### 3.3.3. Rainfall

Changes in rainfall amount, intensity, and frequency caused by climate change will affect atmospheric humidity and soil moisture, while the abundance and infectivity of plant pathogens are significantly influenced by relative humidity and soil moisture. Therefore, climate-related changes in rainfall patterns can impact the occurrence of plant diseases [92]. For example, pathogens that cause soil-borne diseases, such as *Fusarium*, *Rhizoctonia*, *Sclerotium*, *Pythium,* and *Phytophthora*, increase their activity when exposed to excess moisture [93,94]. Fungal pathogens that cause lettuce soft stems have been reported to be more likely to occur when humidity is high [95]. However, there are also other fungi better adapted to drought environments. For example, diseases like pea root rot (*Aphanomyces euteiches*), onion white rot (*Sclerotium cepivorum*), and grapevine black foot (*Ilyonectria/Dactylonectria* spp.) become more severe with longer and more frequent drought conditions [96].

Overlapping rainfall directly affects certain pathogens and insects that overwinter in the soil. Heavy rainfall can result in flooding and prolonged stagnation of water, threatening their survival and diapause [91]. In addition, heavy rains and flooding can wash away insect eggs, larvae, and small-bodied pests like aphids and mites.

#### 3.3.4. pH and Salinity

Soil pH and salinity modulate the incidence and severity of diseases and pests in fruit and vegetable systems by altering host plant physiology and soil microbial communities. Saline conditions (EC > 6 dS/m) weaken root systems and reduce synthesis of defense compounds, increasing susceptibility to soil-borne pathogens like Fusarium and Rhizoctonia [43]. For instance, in tomato, salinity stress enhances infection by Fusarium oxysporum, leading to a 30–40% increase in wilt incidence [79]. Alkaline soils (pH > 7.5) favor the survival of pathogens such as Plasmodiophora brassicae, causing clubroot in cruciferous vegetables [80]. Conversely, acidic soils (pH < 5.5) can suppress certain pathogens like Streptomyces scabies but may increase the virulence of others, such as Phytophthora [34]. Salinity also influences insect behavior: in pepper, high soil salt levels reduce leaf nitrogen content, prompting increased feeding by aphids and whiteflies [84]. Moreover, saline stress disrupts the rhizosphere microbiome, diminishing beneficial microbes that compete with or antagonize pathogens [12]. These interactions emphasize the need for integrated soil management to disrupt disease cycles and enhance plant resilience.

#### 3.3.5. Wind

Wind plays a multifaceted role in the dissemination and proliferation of pests and diseases in horticultural systems. It serves as a primary vector for the long-distance dispersal of fungal spores, such as those of Puccinia and Venturia, and bacterial cells like Xanthomonas [68]. For example, in citrus orchards, wind facilitates the spread of Xylella fastidiosa, a bacterium causing citrus variegated chlorosis, particularly during storm events [97]. Wind-driven rain can also splash soil-borne pathogens like Phytophthora onto low-hanging fruits, increasing rot incidence [92]. In addition, strong winds (>7 m/s) cause physical injuries to plant tissues, creating entry points for wound-invading pathogens such as Botrytis cinerea in strawberries and grapes [81]. Wind stress alters plant morphology and chemistry, often reducing cuticle thickness and increasing leaf nitrogen, which attracts pests like aphids and mites [91]. Furthermore, wind interferes with the efficacy of natural predators and parasitoids by disrupting their foraging behavior, thereby reducing biological control [98]. These dynamics necessitate the use of windbreaks, resistant cultivars, and timely monitoring to mitigate wind-aided pest and disease spread.

#### 3.3.6. UV Radiation

UV radiation has been associated with improving disease resistance in fruits and vegetables by inducing defense-related compounds. For example, in zucchini [99], UV-B treatment alleviated oxidative stress and membrane damage during cold storage, which are frequently associated with heightened susceptibility to post-harvest pathogens. The improvement of antioxidant capacity and flavonoid content might contribute to enhanced cell wall integrity and decreased pathogen invasion. In pepper [69], UV-B—triggered anthocyanin accumulation is part of a wider defense mechanism, as anthocyanins and other flavonoids have antimicrobial and antifungal characteristics. The activation of the CaMYB113—mediated pathway under UV-B stress not only controls pigmentation but may also prepare the plant’s immune response against biotic stress factors.

Unsuitable or excessive UV radiation may also exert a negative influence on the pest and disease resistance of fruits and vegetables and even directly worsen diseases. High doses of UV-B are an environmental stress element. If not managed properly, it can cause lipid peroxidation of plant cell membranes, DNA harm, and a decline in photosynthetic efficiency, thus weakening the overall vigor of plants [100]. This physiological harm will consume a large quantity of metabolic resources used for defense responses, making the fruit and vegetable tissues more fragile and more prone to infection by saprophytic or weakly pathogenic pathogens, resulting in secondary ailments. Additionally, the physical harm caused by UV (such as epidermal burns and tissue depressions) offers direct invasion routes for pathogens, significantly increasing the infection risk. In some situations [101], specific metabolic pathways induced by UV stress may consume the precursors originally utilized for synthesizing other crucial defense compounds (such as lignin or certain phytohormones), leading to a breakdown of the defense system.

#### 3.3.7. Ozone

Long-term exposure to high levels of O_3_ can intensify plant stress, possibly raising the vulnerability to pathogens and pests. Even though it was not directly measured in the given studies, O_3_—induced oxidative stress—shown by the increased malondialdehyde (MDA) levels in radish leaves under high O_3_ (140 ppb)—can damage cellular membranes and undermine plant defense mechanisms, making crops more likely to suffer from secondary infections and insect assaults [74]. This indirect impact may boost pre-harvest losses, especially in sensitive plant varieties. On the bright side, ozone is well-known for its potent antimicrobial and fungicidal characteristics when used after harvest. In stored cucumbers, ozone treatment (10 mg·kg^−1^ for 30 min) notably curbed mold growth and other microbial invasions, thus prolonging the shelf life and preserving product quality for up to 22 days without refrigeration. In contrast, control samples showed obvious spoilage and mold formation by day 20 [102]. The capacity of ozone to quickly break down into oxygen without leaving harmful residues makes it an environmentally friendly substitute for chemical fungicides and pesticides. Furthermore, research suggests that ozone can decrease pesticide residues on agricultural products, as seen in studies with raspberries [103]. Therefore, while the increasing ambient O_3_ may increase disease risks in the field, targeted post-harvest ozone applications present a sustainable approach for managing post-harvest diseases and lessening reliance on synthetic agrochemicals.

#### 3.3.8. Ocean Acidification

The impact of ocean acidification (OA) on the occurrence and spread of plant diseases and pests is an unexplored but crucial area. The main mechanism of its influence lies in altering the overall environment of agricultural ecosystems. In a high CO_2_ environment, the carbon-nitrogen ratio of plants will change, and the nitrogen content in their tissues will decrease relatively. This may cause herbivorous pests [104] (such as aphids, mites, and some lepidopteran larvae) to increase their feeding volume to meet their nitrogen requirements, thereby exacerbating the physical damage to fruits and vegetables. Changes in the chemical composition of plant tissues may also affect the synthesis of their defense compounds, potentially making certain crops more susceptible or resistant to specific pathogens [105] (such as fungi and bacteria), and these changes vary depending on the crops and pathogen types, being highly uncertain. Moreover, OA may change the geographical distribution range of pests and diseases by intensifying climate change. Increased winter temperatures enable pests and pathogens that were unable to survive in cold regions to survive and colonize [68], while changing precipitation patterns may create a more favorable microclimate for the spread and infection of pathogen spores. This expansion of the distribution range will introduce new pest and disease threats into previously safe fruit and vegetable production areas, forcing growers to adjust their integrated pest management strategies, which may increase the frequency and intensity of pesticide use, and trigger new environmental problems.

### 3.4. Impacts of Global Climate Change on Food Safety of Fruit and Vegetable Products

The impacts of climate change on food safety mainly encompass pesticide residue and mycotoxin contamination. Pesticides play a very important role in the prevention, mitigation, and destruction of insect infestation and diseases, while climate change may potentially affect the utilization of pesticides as well as the volatilization and degradation processes in the environment [106]. Additionally, climate represents the key factor in driving the fungal community structure and mycotoxin contamination; thus, climate change could lead to an unexpected increase in relative risk of mycotoxin contamination. Both pesticide residue and mycotoxin contamination could result in serious issues in food safety.

#### 3.4.1. Temperature

Elevated temperatures are conducive to overwintering and reproduction of most pathogens and insects, and increase the risk of invasive alien insect species, which lead to the expansion of plant diseases and agricultural pests and the aggravation of their damage [107]. To control the increasing diseases and pests, coupled with the breakdown of chemical components caused by high temperatures, no doubt fungicides and pesticide usage will increase even more. Furthermore, enhanced pest reproduction rates would result in more severe plant damage and facilitate greater infection by mycotoxigenic fungi, leading to increased mycotoxin contamination. Thus, global warming could further contribute to the increase in mycotoxin contamination [97]. The most important mycotoxigenic fungi infecting fruits and vegetables mainly belong to the genera *Aspergillus* and *Penicillium*, which produce mycotoxins of greatest concern worldwide: aflatoxins (AFs), ochratoxins, and patulin. Reference [108] reported that thermotolerant species will be more advantageous in warmer climates, resulting in the dominance of *Aspergillus* sp. rather than *Penicillium* sp. Furthermore, climate change and variability in the environment may also have an impact on the occurrence of food safety hazards at different stages of the food chain (handling, processing, transporting, trading, etc.). For instance, a temperature rise will increase the risk of food contamination and spoilage in the absence of a cold chain [67].

#### 3.4.2. CO_2_

Elevated CO_2_ levels can increase mycotoxin production by *Aspergillus* and *Fusarium* species through increasing fungal colonization. A study investigated the impact of increased CO_2_ levels (750 ppm CO_2_) on the resistance of wheat against *Fusarium culmorum* [109], along with the occurrence of mycotoxin contamination in various products. Their results indicate that changes in temperature and atmospheric CO_2_ may enhance the risk of *Fusarium* infection and higher mycotoxin contamination under favorable conditions. However, though elevated CO_2_ levels resulted in 2.5 times *Fusarium* biomass production in specific conditions, it had no impact on mycotoxin levels [110]. Maize for stalk rot experiments was grown in two identical environmental Conviron E15 (Pembina, ND, USA) growth chambers controlled at 28 °C day/25 °C night, 500 μmol m^−2^ s^−1^ photosynthetic photo flux density, 12 h photoperiod and between 50 and 60% relative humidity. CO_2_ concentrations were 400 and 800 μmol CO_2_ mol^−1^ air (1 × [CO_2_]) in different growing environments, which is the global atmosphere projected to reach between 2080 and 2100.

#### 3.4.3. Rainfall

Humid conditions following heavy rainfall or floods increase the food safety risk caused by pesticides and mycotoxin contamination. The change in humidity caused by rainfall can affect toxigenic mold colonization and mycotoxin production [111]. In 2017–2018, 150 random mating-derived lines were sown at four sites to enhance the design, and at each site, the experiment was planted when the rains began in June. All recommended crop production and protection practices to develop crops with good plants were followed [112]. They also argue that the genetic material in this study merits further genomic research and marker development for mold tolerance. In another survey, between 2015 and 2017, 150–180 apples per year were collected from each of eight orchards (cv. ‘Ingrid Marie’) in Sweden’s southernmost province of Scania during the regular harvest period from late September to mid-October. Data on apples were collected at harvest and after 1 and 3 months of storage, including fruit weight, firmness level, starch index and lesion size of the fruit [113]. They found that in early June of each year, apples with humidity higher than 77 percent were more likely to get gray mold. During the low temperature and high humidity of August and the end of September, during the end of the fruit cell enlargement stage, correlated with larger apples. In addition, a study based on the effect of early harvesting of peanuts under drought stress in 2018–2019 and 2019–2020 on aflatoxins demonstrated a statistically higher percentage under severe stress (0.4%) compared to non-stress conditions (0.2%). The contamination range of *Aflatus* in soil was 2.52 × 10^3^–1.64 × 10^4^ CFU/g, and the concentration of *Aflatus* in mild and severe drought stress samples was significantly higher than that in control samples. Similarly, [114] also reported a higher prevalence of *Aspergillus* spp. in drier areas of Makueni compared to humid regions.

#### 3.4.4. pH and Salinity

Soil pH and salinity indirectly influence the food safety of fruits and vegetables by affecting the survival and toxin production of contaminating microorganisms. Alkaline conditions (pH > 7.5) can enhance the persistence of human pathogens like E. coli and Salmonella in soil, increasing the risk of preharvest contamination of leafy greens and root vegetables [43]. Saline stress (EC > 8 dS/m) has been shown to promote the production of mycotoxins such as aflatoxin by Aspergillus flavus in peanut and maize, as the fungus responds to osmotic stress by upregulating toxin synthesis [108]. In tomato, soil salinity exacerbates Alternaria infection, leading to higher alternariol mycotoxin levels in fruits [86]. Acidic soils (pH < 5.0) can increase the mobility of heavy metals like cadmium and lead, which may be taken up by crops such as lettuce and spinach, posing a chemical hazard [115]. Additionally, salinity alters the structure of phyllosphere microbial communities, potentially reducing competitive exclusion of foodborne pathogens [79]. These findings highlight the need for soil testing and amendments to manage pH and salinity, thereby reducing microbial and chemical risks in the food chain.

#### 3.4.5. Wind

Wind contributes to food safety risks in fruit and vegetable production by facilitating the dispersal of microbial contaminants and chemical residues. Dust and soil particles carried by wind can deposit pathogenic bacteria (e.g., *Listeria*, *Salmonella*) and fungal spores (e.g., *Aspergillus*, *Penicillium*) onto crop surfaces [116]. For instance, in leafy green production, wind-driven dust from adjacent livestock areas has been linked to E. coli O157:H7 outbreaks [93]. Wind also influences pesticide drift, leading to uneven residue distribution and potential over-application on non-target crops, which may exceed maximum residue limits (MRLs) [117]. In orchards, high wind speeds during spraying can reduce droplet deposition, compromising pest control and necessitating reapplication, thereby increasing chemical load [108]. Moreover, wind-induced fruit scarring and cracking provide niches for mycotoxigenic fungi such as Aspergillus and Fusarium, which can produce aflatoxins and fumonisins under favorable conditions [108]. Postharvest, wind during open-air drying of fruits like figs and dates can introduce contaminants if not properly managed [114]. These risks underscore the importance of wind barriers, buffer zones, and protected cultivation to minimize contamination.

#### 3.4.6. UV Radiation

UV treatment is an effective non-thermal and chemically residue-free intervention measure that can directly inhibit or kill pathogenic microorganisms on the fruit surface, thereby reducing the incidence of post-harvest diseases, pre-harvest UV-B treatment on strawberries [71] significantly reduced the rot rate during storage (mainly caused by Botrytis), thereby extending the shelf life and reducing the food safety risks such as mycotoxins caused by fungal contamination. More importantly, UV radiation can induce the upregulation of the plant’s own defense system and secondary metabolic pathways, thereby indirectly enhancing the safety of the product. Studies [70,71] have shown that UV-B radiation can significantly promote the biosynthesis of phenolic, flavonoid, and anthocyanin substances with antibacterial and antioxidant activities in fruits such as peaches, strawberries, and grapes.

However, the “dose effect” of UV treatment is of crucial importance. An insufficient dose may not effectively inhibit pathogenic bacteria, while an excessive dose may cause physical damage to the fruit (such as burning, degradation of cell membranes), which instead provides a channel for the invasion of pathogenic bacteria, thereby increasing the risk of spoilage and having a negative impact on food safety [118]. In the initial stage of treatment, some secondary metabolites in the peach flesh will temporarily decrease, indicating that the plant may have a metabolic adjustment period when responding to UV stress. During this period, the defense ability may be temporarily weakened [119]. Moreover, if the reactive oxygen species induced by UV accumulation excessively and exceed the cell’s clearance capacity, it will lead to oxidative stress, not only accelerating the deterioration of quality but also potentially affecting the safety of the product.

#### 3.4.7. Ozone

Ozone’s strong oxidative capacity makes it a powerful tool for enhancing food safety. It effectively inactivates a wide spectrum of foodborne pathogens, such as E. coli O157:H7, *Salmonella* spp., and Listeria monocytogenes, on various produce items, including lettuce, strawberries, and peppers, by damaging microbial cell membranes, proteins, and nucleic acids [120,121]. This antimicrobial action is evident in postharvest treatments, where gaseous ozone significantly reduces total microbial growth on kiwifruit, thereby decreasing spoilage and potential health risks [122]. Furthermore, ozone can degrade certain pesticide residues on produce like strawberries and peppers, reducing chemical contaminants [123,124]. A key advantage is that ozone decomposes into oxygen, leaving no harmful chemical residues on the food product, which aligns with organic standards and reduces reliance on chlorine-based sanitizers that can form toxic by-products (FDA, 2001). Studies on tomato irrigation with ozonated water also confirm that it does not lead to the accumulation of phytotoxic elements in the edible parts, supporting its compatibility with safe agricultural practices [125].

However, the efficacy and safety of ozone applications are not without challenges. Its effectiveness can be compromised by factors such as organic matter in water, the surface roughness of produce, and low humidity, which can shield microorganisms and reduce ozone’s stability and penetration [126,127]. Moreover, if misapplied with prolonged or high-concentration exposure, ozone can induce oxidative stress in the plant tissues themselves, potentially affecting metabolite profiles and leading to quality loss [122]. There is also a potential, though less concerning than with chlorine, for ozone to react with organic compounds to form secondary oxidation products. Therefore, while ozone is a viable and sustainable option for enhancing the microbial and chemical safety of fresh produce, its deployment requires precise optimization of concentration, exposure time, and environmental conditions to maximize benefits and minimize any adverse effects on both food safety and quality.

#### 3.4.8. Ocean Acidification

The potential risks posed by ocean acidification (OA) to the safety of fruit and vegetable products are indirect and complex, mainly achieved through the influence on the behavior of environmental pollutants and the microbial ecology. One of the most concerning pathways is the bioaccumulation of heavy metals. OA can alter the chemical forms of heavy metals (such as cadmium, lead, and mercury) in the ocean, making them more bioavailable [128]. After these heavy metals are absorbed by marine organisms, they may be transmitted through the food chain. When fish meal made from seafood or irrigated with affected coastal seawater is used, there is a risk of these heavy metals entering the terrestrial food chain and accumulating in the soil, ultimately being absorbed by fruit and vegetable crops [129], leading to excessive heavy metal content in the products and posing long-term health risks. Secondly, the interaction between OA and climate change may affect the contamination of mycotoxins. Changes in temperature and humidity conditions may promote the growth and toxin production of certain toxigenic fungi [130] (such as *Aspergillus* species that produce *aflatoxins* and *Penicillium* species that produce ochratoxins) on fruit and vegetable products in the field or during storage. For example, during the harvest period, when there is heavy rainfall and the temperature is suitable, the risk of fruit (such as grapes and nuts) being infected by fungi and accumulating toxins will significantly increase. These mycotoxins are strong carcinogens and poisons [131], posing a serious threat to food safety. Therefore, although ocean acidification does not directly pollute fruit and vegetable products, as an environmental stress multiplier, it potentially raises the background risk level for the safety of fruit and vegetable products by altering the pollutant cycle and the microbial environment.

### 3.5. Discussion on the Systemic Impact of Climate Factors on the Fruit and Vegetable Industry

The cumulative evidence underscores that the impact of global climate change on the fruit and vegetable industry is not merely a sum of isolated stressors but a complex, systemic phenomenon with cascading effects across the entire production and post-production continuum. The individual climatic factors—temperature, CO_2_, water variability, soil properties, wind, UV radiation, and tropospheric ozone—interact in ways that can amplify risks and create novel challenges, fundamentally threatening the sector’s stability, product integrity, and safety.

The most direct systemic impact is on yield stability and nutritional security. Rising temperatures drive substantial yield losses in key crops like pineapple, tomato, and peach by disrupting critical physiological processes such as pollination, fruit set, and development [23,24,26]. While elevated CO_2_ can potentially stimulate photosynthesis and mitigate water stress in some cases (e.g., tomato [28,29]), it often comes at the cost of reduced nutritional density, altering the mineral and protein content of edible parts [63,64]. This creates a critical trade-off between quantity and quality, directly impacting human nutrition. Furthermore, water extremes with both droughts and excessive rainfall compromise yield by causing physiological drought, fruit drop, and root damage [30,40], while soil salinity and pH shifts induce osmotic stress and nutrient disorders, leading to linear yield declines, as starkly demonstrated in date palm [32].

Beyond yield, climate change acts as a pervasive force redefining product quality and market value. High temperatures alter the delicate balance of sugars, acids, pigments, and antioxidants, leading to apples with poor color and peaches with reduced sweetness [33,36]. Altered precipitation patterns can result in watery, bland-tasting fruits like guava [37]. These shifts not only diminish consumer acceptance but also affect the processing quality and shelf life of produce. Notably, factors like UV-B radiation and ozone present a “double-edged sword”; while moderate UV-B can enhance beneficial compounds like anthocyanins and antioxidants in peppers and grapes [69,70], excessive exposure causes photoxidative damage and quality deterioration [49].

Perhaps the most insidious systemic impact lies in the alteration of pest and disease dynamics and food safety. Climate change disrupts the delicate ecological balance between crops, their pathogens, and pests. Warmer temperatures expand the geographical range of pests and pathogens, extend their reproductive seasons, and increase their reproductive rates [80,84]. Elevated CO_2_ can weaken plant defense mechanisms, making them more susceptible to infections and leading to higher consumption rates by insects to compensate for lower tissue nitrogen [89,91]. Changes in humidity and rainfall patterns create microenvironments that favor soil-borne diseases and fungal pathogens [92,95]. This escalated pressure directly translates into heightened food safety risks. The increased prevalence of mycotoxigenic fungi like *Aspergillus* and *Fusarium*, driven by warmer and more humid conditions, elevates the risk of *mycotoxin* contamination [97,108]. Moreover, the increased pesticide usage required to control these burgeoning pest and disease populations raises concerns about chemical residue levels on fresh produce [106].

In conclusion, the climate factors reviewed do not operate in isolation. A heatwave can simultaneously reduce yield, impair quality, weaken plant defenses, and encourage pathogen growth, while associated drought or heavy rainfall further compounds these effects. This systemic interplay creates a “perfect storm” of challenges that threatens the very foundation of a secure, safe, and nutritious fruit and vegetable supply. Addressing these interconnected risks requires a move beyond single-factor management towards integrated, system-level resilience strategies.

## 4. Impacts of Carbon Pricing Policies on the Fruit and Vegetable Industry

**Figure 2:**
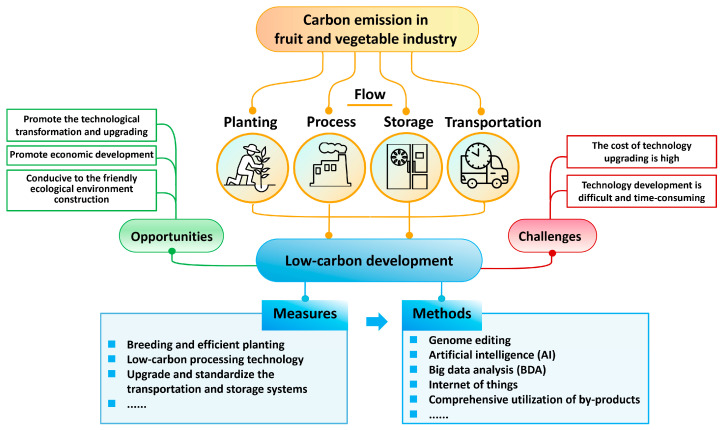
Strategies, opportunities and challenges for low-carbon development of the fruit and vegetable industry.

The fruit and vegetable industry, despite having a relatively low carbon footprint compared to animal-based foods [132], is not exempt from the pressures of carbon pricing. Its emissions are inherently linked to input-intensive cultivation practices (e.g., synthetic fertilizers, pesticides) and energy-dependent post-harvest processes (e.g., refrigeration, transportation, processing) (Figure 2). Consequently, the implementation of carbon pricing policies sends price signals throughout this supply chain. The subsequent rise in costs for fossil-fuel-based inputs and energy creates a direct economic incentive for emission reduction [133]. However, the transmission of these costs and the adaptive capacity of market participants vary considerably. For example [134], large-scale, vertically integrated producers may absorb costs or invest in low-carbon technologies (e.g., renewable energy for cold storage), whereas smallholder farmers often experience disproportionate financial strain, potentially worsening equity concerns. This dynamic affects not only production and distribution patterns but also initiates a systemic shift, necessitating a re-evaluation of operational efficiencies, supply chain logistics, and ultimately, encouraging the sector’s transition towards a low-carbon future through technological innovation and structural adaptation.

### 4.1. Impacts of Carbon Pricing Policies on the Supply Chain

#### 4.1.1. Planting

The planting stage constitutes a significant source of carbon emissions in the fruit and vegetable industry, primarily driven by the production and application of chemical fertilizers and pesticides [135]. A life cycle assessment (LCA) of orchards in China quantified this impact, revealing that nitrogen fertilizer inputs alone account for 47–75% (93–204 kg CO_2_-eq/ton of fruit) of total GHG emissions up to the point of harvest [136]. This high share underscores the sector’s vulnerability to policies that price carbon, as the manufacturing of these agro-inputs is highly energy-intensive.

Carbon pricing policies exert their influence on planting activities through a direct cost-transmission mechanism. By increasing the cost of fossil fuels, these policies raise the production costs of fertilizers and pesticides, which are subsequently passed down to farmers [137]. This surge in input costs alters the economic calculus of production. Faced with squeezed profit margins, farmers are incentivized to adapt their practices. These adaptations can range from input substitution (e.g., replacing synthetic fertilizers with organic alternatives), precision agriculture (e.g., using sensor-based technologies to optimize application rates and timing), to more drastic measures like crop diversification or reduction in planted area for the most input-intensive crops [138].

An evaluation of the U.S. apple industry illustrates this complex trade-off: while a carbon tax effectively reduced GHG emissions, with higher tax rates yielding greater mitigation, it also led to a decline in apple production [139]. This outcome highlights a critical policy dilemma: the tension between environmental efficacy and production levels. The extent of this trade-off is not uniform; it is mediated by farmers’ access to capital, knowledge, and low-carbon technologies. Without concomitant support systems, carbon pricing risks disproportionately burdening smallholders who lack the capacity to invest in efficiency gains [140], potentially leading to inequitable outcomes and threatening localized food supply.

#### 4.1.2. Distribution

Throughout the entire distribution system, energy consumption is one of the primary sources of carbon emissions. Taking the export of Spanish tomatoes to several European countries as an example [141], the transportation aspect of the supply chain, including the geographical separation of production, processing, and export hubs, has significantly increased overall energy consumption through “recycling transportation.” Research indicates that in some export-oriented provinces, transportation-related energy consumption can account for more than 13% of the entire chain. Among these, land transportation and cross-border logistics consume considerable amounts across various product categories, and the cold chain link, due to the necessity of maintaining product freshness, has also become a significant source of energy consumption. For instance [142], in target markets such as the United Kingdom, the carbon emissions from food refrigeration have accounted for approximately 2% to 3% of the country’s total emissions, highlighting the critical role of the cold chain system in the overall carbon footprint. With the gradual implementation of carbon pricing mechanisms, there has been a significant impact on the distribution strategies of fruits and vegetables. When the carbon price surpasses a certain threshold, supply chain entities tend to optimize transportation routes, reduce reliance on long-distance cold chains, and even adjust product portfolios. For example [143], favoring products that are easier to store, local, or seasonal. This transformation affects not only logistics decisions but may also extend to the production end, leading to changes in regional planting structures. This reflects the important regulatory role of carbon policies in guiding the low-carbon transformation of the agricultural system.

The distribution of fruit and vegetable products, encompassing harvesting, processing, storage, and transportation, constitutes an energy-intensive and significant source of carbon emissions within the supply chain. A lifecycle assessment of the avocado supply chain from South Africa to Switzerland vividly illustrates the emission structure, with road (45.9%) and maritime transport (48.3%) being the dominant contributors, while storage and handling operations account for a smaller share (5.8%) [144]. This emission profile underscores the distribution system’s acute exposure to carbon pricing policies.

Carbon pricing policies fundamentally reshape logistics decision-making by internalizing the previously externalized cost of emissions [145]. As the carbon price rises, it directly increases operational expenses for fuel, electricity for cold storage, and refrigeration. Logistics firms, being highly sensitive to such cost signals [146], are compelled to optimize their networks beyond traditional efficiency metrics. This triggers a multi-faceted strategic response: (1) Supply chain shortening and localization, where companies may nearshore production or prioritize regional markets to reduce long-haul transportation emissions [147]; (2) Modal shift, favoring lower-emission transport options (e.g., rail over road for land transport) where feasible [148]; and (3) Technological and operational innovation in cold chains, such as investing in energy-efficient refrigeration systems or leveraging AI for dynamic temperature control to reduce energy consumption [149].

These operational shifts have profound downstream implications for product portfolios and, consequently, upstream planting structures. A higher implicit carbon cost makes the distribution of easily stored, local, and seasonal products more economically attractive relative to energy-intensive, globally traded fresh produce. This changing demand signal may eventually discourage the cultivation of crops destined for long-distance, refrigeration-heavy supply chains, thereby influencing farmers’ planting decisions. This dynamic illustrates a critical, albeit indirect, pathway through which carbon pricing can induce a systemic transition towards more resilient and less emission-intensive fruit and vegetable systems.

#### 4.1.3. Impacts on Smallholder Farmers and Distributional Equity

While carbon pricing policies are designed to incentivize broad decarbonization, their implementation often imposes a disproportionate financial burden on smallholder farmers, raising critical concerns regarding equity and local food security. Smallholders typically operate with limited capital and economies of scale, making them less able to absorb the increased costs of fossil-fuel-based inputs (e.g., fertilizers, pesticides) and energy for irrigation or post-harvest handling [150]. This cost shock can force difficult trade-offs, potentially reducing input use, shrinking cultivated areas, or even causing farm exit, thereby threatening local and regional supplies of diverse fruits and vegetables [151]. The ensuing reduction in production volume and farmer profitability can exacerbate vulnerabilities in local food systems, particularly in regions where smallholders are key suppliers of fresh produce.

Addressing this inequity requires carefully designed policy sequencing and targeted compensation mechanisms [152]. As indicated in our systems analysis, initial subsidies for low-carbon technologies such as photovoltaic-powered cold chains, energy-efficient irrigation systems, and renewable energy microgrids are crucial to precede or accompany the rollout of carbon taxes. This “support-then-price” approach helps smallholders transition without triggering profit-loss tipping points [153]. Furthermore, revenue recycling from carbon pricing can be directed towards direct payments, conditional grants, or subsidized access to climate-resilient seeds and organic fertilizers for small-scale producers [154]. Such measures not only mitigate immediate financial stress but also enhance adaptive capacity. Without these targeted interventions, carbon pricing risks undermining the livelihoods of vulnerable farmers and the resilience of local food systems [155], thereby conflicting with broader sustainable development goals. Policymakers must, therefore, integrate robust equity assessments and safety nets into climate policy frameworks to ensure a just transition for the fruit and vegetable sector.

### 4.2. Impacts of Carbon Pricing Policies on the Marketing

Carbon pricing policy is not just a regulatory step; it is a highly impactful force in molding the fruit and vegetable market, basically changing marketing tactics from the farm entrance to the retail display. Its influence goes well beyond simple cost transfer and sets off complex reorganizations of produce positioning, consumer interaction, and competitive edges within the fresh produce industry. The early execution of carbon pricing is often regressive [156], and because of the carbon-intensive character of modern horticulture (e.g., greenhouse warming, cooling, and long-distance shipping), it disproportionately impacts the cost frameworks of entire fruit and vegetable supply chains. This distribution result poses serious marketing difficulties for supermarkets and fresh food brands [139]: they have to manage the reputational and commercial risks linked with rising prices of essential items like fresh fruits and vegetables. Nevertheless, when revenues are redistributed nationwide via a per-person “carbon dividend [157]”, the policy can turn progressive. This creates a two-fold marketing necessity for produce retailers: first, to promote fair policy planning to safeguard low-income fresh food consumers [158]; and second, to foresee and meet the changed purchasing power and possibly increased environmental awareness of consumers who might now be more ready to pay extra for low-carbon, locally sourced, or sustainably grown produce [159].

At the micro-level of market rivalry, carbon limitations compel fruit and vegetable businesses to strategically reassess their marketing blend, especially regarding pricing and product distinctiveness [160]. The ideal price for a head of lettuce or a kilogram of apples under a carbon price is affected by the emission efficiency of its production and cold-chain logistics, the allotted carbon quota, and, importantly, the value consumers get from low-carbon features, such as “carbon footprint” labels on produce [161]. When a fruit grower or a vegetable distributor cuts per-unit emissions, for example, by using renewable energy for greenhouses or streamlining transport routes, it can obtain a cost benefit within the quota, possibly resulting in more competitive pricing, a powerful volume-based approach for staple vegetables [162]. On the other hand, a specialty fruit brand or an organic vegetable box program can pursue a premium setting strategy by capitalizing on consumers’ growing preference for low-carbon produce, allowing them to charge higher prices [163]. So, marketing under the carbon pricing mechanism is not a one-size-fits—all defensive approach but a strategic option for produce companies: either becoming a cost leader through operational greening or a distinct competitor through a clear “green produce” brand plan, with the best route depending on the firm’s technological abilities and its evaluation of modern food consumer values.

This link between producer motivations and the consumer market is critically obvious in the horticultural sector. The readiness of fruit farmers and vegetable growers to adopt sustainable practices [164], a central part of the “farm-to-fork” sustainable produce story, is very sensitive to payments for ecosystem services, including potential carbon credits for soil carbon storage and price premiums for certified low-carbon fruits and vegetables [160]. Research shows that small-scale vegetable growers, similar to coffee farmers, are often more averse to the perceived risks and opportunity costs of switching to low-carbon methods and may need more compensation and technical assistance [164]. This emphasizes a key aspect of marketing strategy for fresh produce brands: the sustainability claim on a bag of salad or a bunch of grapes is only as trustworthy as the resilience and fairness of its supply chain. Thus, marketing should not only communicate with end consumers at the supermarket but also involve the design of incentive structures [165] like long-term contracts and cost-sharing agreements to make sure upstream fruit and vegetable farmers adopt sustainable practices, thus securing the “green” characteristic at its origin.

### 4.3. Main Countermeasures to Carbon Pricing Policies and Available Technologies

#### 4.3.1. Main Countermeasures to Carbon Pricing Policies and Challenges

The execution of carbon pricing policies is prompting a strategic reshaping of the whole supply chain of the fruit and vegetable industry from the field to the table. The central strategy for fruit growers and vegetable growers is to shift from conventional farming to accurate and low-carbon agriculture [166], by utilizing renewable energy heating in greenhouses and curbing implicit carbon emissions via precise irrigation and fertilization, etc. For origin distributors and processing companies [167], they confront the hurdle of decarbonizing the post-harvest phase. The crux lies in advancing the electrification of the cold chain system and leveraging big data to optimize storage and logistics routes [168] to reduce the carbon footprint of the “from farm to market” stage.

For large fruit and vegetable retail businesses and brand proprietors, enhancing supply chain energy efficiency is the essence of their carbon competitiveness. This demands that they look beyond their own emissions and set up a carbon accounting model encompassing the entire supply chain [169], precisely monitoring the carbon footprint of each batch of products. A more profound solution is to spearhead the structural integration of the supply chain. By sharing data with contracted farmers and jointly constructing low-carbon cold chain infrastructure, they can bring scattered fruit farmers into a modern emission reduction system, thus boosting the efficiency and resilience of the whole supply network [170]. Meanwhile, astute fruit and vegetable marketers have started to turn carbon costs into market benefits. They actively promote local seasonal products, making “zero storage emissions” and “low transportation mileage” compelling selling points to draw in environmentally conscious consumers [171].

Nevertheless, this transformation is full of different challenges according to the role. The majority of fruit and vegetable farmers, along with small and medium-sized distributors, are on the front line, facing a tough “green conundrum”: the high upfront investment (such as energy-efficient cold storage facilities, electric transport vehicles) makes it hard for them to balance the transition and survival [172]. For large fruit and vegetable enterprises, the challenge is how to conduct expensive low-carbon renovations of their existing heavy-asset cold chain network without disrupting their large and complex supply chain [173]. Ultimately, all these increased costs may be transferred to the end market, testing consumers’ tolerance for high-priced low-carbon fruits and vegetables. If not managed well, it may dampen overall demand [174].

Hence, precise policy intervention is extremely significant. The government should offer special green subsidies and loans to fruit farmers and small and medium-sized distributors, and set a distinct carbon price transition period for the entire industry. This is to prevent carbon pricing from changing from an innovation stimulator to a regressive tool that intensifies market monopolies [175] and damages the vulnerable groups in the industrial chain during its implementation.

#### 4.3.2. The Role of Multilevel Governance and Local Institutions in a Just Transition

To attain a just and low-carbon transformation of the fruit and vegetable industry, relying merely on market forces or policies at a single level is simply insufficient. It highly depends on the collaborative endeavors of multi-level governance systems and local institutions to guarantee that the carbon cost does not unjustly burden the vulnerable groups in the industrial chain [176].

At the national level, the government’s core function is to create a fair, competitive environment and offer strategic support. This entails formulating differentiated carbon pricing rules for the fruit and vegetable industry [177], particularly for small-scale fruit farmers and small and medium-sized distributors. For instance, setting transition periods or providing subsidies for key inputs like fertilizers and cold chain energy to prevent production shrinkage caused by “one-size-fits-all” policies [178]. Meanwhile, a special transformation fund should be set up to offer low-interest loans for fruit farmers to renovate energy-efficient greenhouses and for distributors to buy electric refrigerated vehicles, directly easing the capital obstacles they encounter [179].

Local governments and agricultural cooperatives play an irreplaceable part in the implementation of policies at the “last mile”. They are crucial in translating national policies into local actions [180]. Local governments can take the lead in establishing regional low-carbon fruit and vegetable production and marketing alliances [181], integrating the production resources of local farmers, and negotiating with buyers such as supermarkets and fresh food e-commerce platforms to establish a high-quality and high-price procurement mechanism that reflects the benefits of carbon reduction [182]. Local agricultural cooperatives and technical promotion stations become the core nodes for knowledge spread and technology empowerment [183], providing customized carbon footprint accounting and green planting technology training for scattered farmers, and assisting them in connecting with the carbon credit market, turning ecological practices into tangible extra income.

Local institutions, such as the fruit industry associations and farmers’ cooperatives, are of great significance in building trust and collective action. They can organize scattered fruit farmers and form a scale large enough to attract investment [184], jointly investing in low-carbon infrastructure such as community solar, cold storage facilities and shared electric logistics fleets, which individual farmers cannot afford. Furthermore, these institutions are important mediators in cultivating consumer trust. By establishing localized low-carbon agricultural product certification and traceability systems [185], they offer clear and distinguishable low-emission product choices for end consumers, thus effectively transmitting consumers’ willingness to pay to the production end, forming a sustainable green premium cycle.

#### 4.3.3. Available Technologies for Low-Carbon Development

The energy conservation and emission reduction in the fruit and vegetable industry require the application and innovation of science and technology, involving biotechnology, information technology, internet technology, big data, and other high-tech fields. The following low-carbon technologies are available in the fruit and vegetable industry from the aspects of breeding, cultivation, processing, and transportation.

Using molecular marker-assisted breeding [186] and transgenic or genome editing breeding techniques [187], new fruit and vegetable varieties can have higher photosynthetic efficiency or better growth under biotic and abiotic stresses, and present higher yield and quality. In the Mediterranean region, kaolin, as a sustainable anti-transpiration and stress-resistant material, has been proven to effectively alleviate the stress caused by high temperatures and intense sunlight on olive trees. Research [188] shows that after spraying kaolin, the oil content of “Racioppella” olive fruits significantly increased (+3.4%), the proportion of monounsaturated fatty acids (such as oleic acid) in the oil rose, the proportion of polyunsaturated fatty acids decreased, and the total polyphenol content increased by 11%. These changes not only improved the nutritional quality and oxidative stability of the oil but also enhanced its market competitiveness. This technology is simple to operate and environmentally friendly, and is suitable for small-scale farmers in the Mediterranean region to achieve the goal of high-quality and stable production in the context of climate change.

Some low-carbon cultivation technologies have positive effects on the sustainable development of the fruit and vegetable industry. Water-saving irrigation technology based on water-saving irrigation equipment and precision irrigation management can dramatically improve water use efficiency [189]. Three-dimensional cultivation technology, such as plant factories, can achieve higher yields and lower GHG emissions through improving land-use efficiency [190]. The indoor vertical farming system provides fruits and vegetables year-round without being affected by climate factors [191]. Despite its considerable initial investment, which includes the costs of structures, sensors, lighting, and water supply systems, the payback period is usually three to five years. However, the integration of renewable energy, modular design, and efficient monitoring systems is gradually reducing these obstacles [191]. Organic agricultural technology can reduce carbon emissions by sequestering carbon in soil and decreasing the use of chemical pesticides and fertilizers [66]. Organic agricultural technology can provide high-quality and safe products, but the yield needs to be further improved by combining with breeding and other cultivation technologies.

In terms of fruit and vegetable processing, the integrated utilization efficiency of by-products should be increased, which can decrease agricultural waste. For example, the development of mycelium biocomposite materials that can utilize different agricultural residues [192], and the use of fruit and vegetable processing by-products to manufacture degradable food packaging [193], have gradually become a trend in recent years.

In storage and logistics, the low-carbon cold chain logistics of fresh fruit and vegetable products is the most effective way to reduce carbon emissions. Postharvest storage losses in agriculture range from 15–20%, of which 5–10% for fruits, due to unreasonable storage parameters and logistics distribution systems [194]. In recent years, with the progress of information technologies such as the Internet of Things, blockchain, artificial intelligence (AI), and big data analysis (BDA), the digitization process of the cold chain has been accelerated, providing more realistic algorithms and strategies for the cold chain of fresh agricultural products and their packaging forms [195].

Low-carbon technologies also bring challenges to rural areas and farmers. The development of technology depends on the construction of infrastructure [196], but rural infrastructure construction is limited by financing opportunities, low local mass acceptance, high construction costs, technological backwardness, lack of subsidy policy, other infrastructure and professional talents, insufficient government funding, etc. [197]. In the process of applying low-carbon technology in rural areas, financial investment in planning, operation and maintenance of rural compulsory education should be increased, rural financing models should be expanded, and more talents and funds should be introduced.

## 5. Conclusions

This review systematically examines the challenges and future paths faced by the fruit and vegetable industry in the context of global climate change and policy transformation.

At the impact level, the research reveals that climate change poses a comprehensive and systematic threat to the yield, quality, pest and disease occurrence, and food safety of the fruit and vegetable industry through various factors such as temperature, CO_2_, water, soil, ultraviolet rays, and even ground ozone. These impacts do not exist independently but exhibit complex interactions, and their effects vary depending on crop types and geographical regions, highlighting the complexity and context-dependency of response strategies. At the policy level, low-carbon policies represented by carbon pricing inject impetus for emission reduction in the industry while also bringing significant distributional challenges. The research finds that carbon costs are transmitted along the entire supply chain from “production-distribution-marketing”, but their impact is not balanced: small farmers bear disproportionate pressure due to limited capital and technology; distributors are forced to optimize logistics networks to reduce emission costs; and retailers need to make fundamental adjustments to marketing strategies to convert carbon costs into market advantages. To address these challenges, a multi-level governance system with precise intervention is required: the role of the national government is to build a fair top-level policy framework and provide strategic financial support; while local governments and local institutions (such as agricultural cooperatives) play an irreplaceable role in promoting the implementation of policies at the “last mile”, organizing scattered farmers, and building regional low-carbon production and sales alliances. In terms of solutions and highlights, the core argument of this paper is that policies, technologies, and governance must be strategically coordinated rather than simply layered. Although low-carbon technologies such as genome editing, water-saving irrigation, vertical agriculture, organic cultivation, and digital cold chain provide a powerful toolkit for enhancing industry resilience and efficiency, their widespread application is not a spontaneous process. The large-scale promotion of technologies relies on forward-looking policy guidance and infrastructure investment. Particularly important is the policy sequence: prioritizing investment in renewable energy infrastructure (such as providing subsidies for agricultural solar energy) is a key prerequisite for avoiding profit loss thresholds and unlocking the potential for technological emission reduction.

In conclusion, building a “resilient” fruit and vegetable industry that can withstand climate shocks and achieve a low-carbon transformation is a complex project. This requires us to go beyond traditional thinking and promote interdisciplinary knowledge integration, cross-departmental policy coordination, and cross-border action collaboration. Only through such systematic integration can we ensure the sustainability of the key fruit and vegetable industry in the context of global change, and thereby safeguard global nutritional security.

## Figures and Tables

**Table 1:** Impacts of climate factors on fruit and vegetable yield and quality.

Aspects	Climate Factors	Fruit and Vegetable Categories	Effects	Mechanisms	Location	References
Yield	Temperature	Pineapple	Decrease by 6% for every 1 °C change	Extremely high temperature affects flowering	Fields in Queensland, Australia	[23]
		Japanese apricot	Decrease by 100 kg/1000 m^2^ for every 1 °C increase	Temperature affects pollination and fruit setting	Fields in Wakayama, Japan	[25]
		Peach	Decrease	High temperature affects flower blooming and fruit development	Fields in Bordeaux, Balandran, Étoile-sur-Rhoône, France	[26]
			Decrease	Abnormal winter temperature affects flowering and fruit setting	Fields in Mornag, Tunisia	[27]
		Tomato	Decrease by 70%	Pollen quality and viability were poor at high temperatures	Fields and greenhouses in Cambodia	[24]
	CO_2_	Tomato	Increase	Higher CO_2_ concentration facilitates the transfer of photosynthetic products to fruit	Controlled environmental chambers in Zhenjiang, China	[28]
		Cherry tomato	Yield increase under reduced irrigation	Elevated CO_2_ improves root development and nitrogen uptake and increases irrigation water productivity	Controlled environmental chambers in Wuwei, China	[29]
		Dragon fruit	Decrease	Higher rainfall causes the drop of dragon fruit buds or flowers, and fruit decay	Fields in Baramati, India	[30]
		Loquat	Decrease	Frequent rainfall affects pollinator activity	Fields in Ras Munif, Jordan	[31]
	Soil pH and salinity	Palm	Decrease by 44% and 64% at 10 ds/m and 15 ds/m	Salinity stress	Fields in Dubai, United Arab Emirates	[32]
Quality	Temperature	Apple	Decrease in soluble solids content, increase in titratable acidity, abnormal skin color	High temperature decreases the leaf photosynthetic rate and affects anthocyanin accumulation	Controlled environmental Facility in Korea	[33]
		Tomato	High temperature changes mineral content depending on the cultivar	N/A	Greenhouse in Texcoco, Mexico	[34]
		Strawberry	Increase in antioxidant activity	High temperature increases the total polyphenol content	Controlled environmental chambers in Melbourne, Australia	[35]
		Peach	Decrease in fruit weight, size, and sweetness	High temperatures retard late fruit development	Controlled environmental chambers in Kagawa, Japan	[36]
	CO_2_	Cherry tomato	Increase in contents of soluble solids, vitamin C, and lycopene	High CO_2_ level increases photosynthesis	Controlled environmental chambers in Wuwei, China	[29]
	Rainfall	Guava	Decrease in texture and sugar content	Excessive rainfall during fruit development reduces the accumulation of photosynthetic products	N/A	[37]

## Data Availability

No new data were created or analyzed in this study. Data sharing is not applicable to this article.

## References

[1] McCulloch M.T., Winter A., Sherman C.E., Trotter J.A. (2024). 300 years of sclerosponge thermometry shows global warming has exceeded 1.5 °C. Nat. Clim. Change.

[2] Wang F., Harindintwali J.D., Yuan Z., Wang M., Wang F., Li S., Yin Z., Huang L., Fu Y., Li L. (2021). Technologies and perspectives for achieving carbon neutrality. Innovation.

[3] Cheng H. (2020). Future Earth and Sustainable Developments. Innovation.

[4] Semba R.D., Askari S., Gibson S., Bloem M.W., Kraemer K. (2022). The Potential Impact of Climate Change on the Micronutrient-Rich Food Supply. Adv. Nutr..

[5] Ou L., Zhang Y., Zhang Z., Chen Y., Wang K., Wen Y., Ao Y. (2023). The relationship between canopy microclimate, fruit and seed yield, and quality in Xanthoceras sorbifolium. J. Plant Physiol..

[6] Springmann M., Sacks G., Ananthapavan J., Scarborough P. (2018). Carbon pricing of food in Australia: An analysis of the health, environmental and public finance impacts. Aust. N. Z. J. Public Health.

[7] Lin T.-Y., Chiu Y.-H., Lin Y.-N., Chang T.-H., Lin P.-Y. (2023). Greenhouse gas emission indicators, energy consumption efficiency, and optimal carbon emission allowance allocation of the EU countries in 2030. Gas Sci. Eng..

[8] Feng X., Zhao Y., Yan R. (2024). Does carbon emission trading policy has emission reduction effect?—An empirical study based on quasi-natural experiment method. J. Environ. Manag..

[9] Jameel S. (2023). Climate change, food systems and the Islamic perspective on alternative proteins. Trends Food Sci. Technol..

[10] Wang R., Bai Z., Chang J., Li Q., Hristov A.N., Smith P., Yin Y., Tan Z., Wang M. (2022). China’s low-emission pathways toward climate-neutral livestock production for animal-derived foods. Innovation.

[11] Zeng X., Wang X., Li J. (2025). Environmental Affects of Plant-Based Beef Analog Production in 12 Countries and Consumer Response to Plant-Based Meat in China, A Case Study. J. Food Nutr. Res..

[12] Pugnaire F.I., Morillo J.A., Peñuelas J., Reich P.B., Bardgett R.D., Gaxiola A., Wardle D.A., van der Putten W.H. (2019). Climate change effects on plant-soil feedbacks and consequences for biodiversity and functioning of terrestrial ecosystems. Sci. Adv..

[13] Chaudhry S., Sidhu G.P.S. (2022). Climate change regulated abiotic stress mechanisms in plants: A comprehensive review. Plant Cell Rep..

[14] Malhi G.S., Kaur M., Kaushik P. (2021). Impact of Climate Change on Agriculture and Its Mitigation Strategies: A Review. Sustainability.

[15] Medda S., Fadda A., Mulas M. (2022). Influence of Climate Change on Metabolism and Biological Characteristics in Perennial Woody Fruit Crops in the Mediterranean Environment. Horticulturae.

[16] Carter P., Gray L.J., Talbot D., Morris D.H., Khunti K., Davies M.J. (2012). Fruit and vegetable intake and the association with glucose parameters: A cross-sectional analysis of the Let’s Prevent Diabetes Study. Eur. J. Clin. Nutr..

[17] Li B., Chen Y., Zhang Z., Qin G., Chen T., Tian S. (2020). Molecular basis and regulation of pathogenicity and patulin biosynthesis in *Penicillium expansum*. Compr. Rev. Food Sci. Food Saf..

[18] Collaborators G.B.D.D. (2019). Health effects of dietary risks in 195 countries, 1990-2017: A systematic analysis for the Global Burden of Disease Study 2017. Lancet.

[19] Stratton A.E., Finley J.W., Gustafson D.I., Mitcham E.J., Myers S.S., Naylor R.L., Otten J.J., Palm C.A. (2021). Mitigating sustainability tradeoffs as global fruit and vegetable systems expand to meet dietary recommendations. Environ. Res. Lett..

[20] Feng S., Lakshmanan P., Zhang Y., Zhang T., Liang T., Zhang W., Chen X., Wang X. (2023). A comprehensive continental-scale analysis of carbon footprint of food production: Comparing continents around the world. J. Clean. Prod..

[21] Salinas I., Hueso J.J., Cuevas J. (2021). Active Control of Greenhouse Climate Enhances Papaya Growth and Yield at an Affordable Cost. Agronomy.

[22] Schmitt J., Offermann F., Soeder M., Fruehauf C., Finger R. (2022). Extreme weather events cause significant crop yield losses at the farm level in German agriculture. Food Policy.

[23] Haque S., Akbar D., Kinnear S. (2020). The variable impacts of extreme weather events on fruit production in subtropical Australia. Sci. Hortic..

[24] Ro S., Chea L., Ngoun S., Stewart Z.P., Roeurn S., Theam P., Lim S., Sor R., Kosal M., Roeun M. (2021). Response of Tomato Genotypes under Different High Temperatures in Field and Greenhouse Conditions. Plants.

[25] Maeda T., Hiraiwa M.K., Shimomura Y., Oe T. (2023). Weather conditions affect pollinator activity, fruit set rate, and yield in Japanese apricot. Sci. Hortic..

[26] Vanalli C., Casagrandi R., Gatto M., Bevacqua D. (2021). Shifts in the thermal niche of fruit trees under climate change: The case of peach cultivation in France. Agric. For. Meteorol..

[27] Ghrab M., Ben Mimoun M., Masmoudi M.M., Ben Mechlia N. (2014). Chilling trends in a warm production area and their impact on flowering and fruiting of peach trees. Sci. Hortic..

[28] Akhlaq M., Chuan Z., Haofang Y., Shaowei L., Ni Y., Zhou J., Xue R., Li J., Hussain Z., Iqbal S. (2023). Exploring adequate CO_2_ elevation for optimum tomato growth and yield under protected cultivation. J. Plant Physiol..

[29] Du B., Shukla M.K., Yang X., Du T. (2023). Enhanced fruit yield and quality of tomato by photosynthetic bacteria and CO_2_ enrichment under reduced irrigation. Agric. Water Manag..

[30] Wakchaure G.C., Minhas P.S., Kumar S., Mane P., Suresh Kumar P., Rane J., Pathak H. (2023). Long-term response of dragon fruit (Hylocereus undatus) to transformed rooting zone of a shallow soil improving yield, storage quality and profitability in a drought prone semi-arid agro-ecosystem. Saudi J. Biol. Sci..

[31] Freihat N.M., Al-Ghzawi A.A.-M., Zaitoun S., Alqudah A. (2008). Fruit set and quality of loquats (Eriobotrya japonica) as effected by pollinations under sub-humid Mediterranean. Sci. Hortic..

[32] Al-Dakheel A.J., Hussain M.I., Abdulrahman A., Abdullah A. (2022). Long term assessment of salinity impact on fruit yield in eighteen date palm varieties. Agric. Water Manag..

[33] Lee I.B., Jung D.H., Kang S.B., Hong S.S., Yi P.H., Jeong S.T., Park J.M. (2023). Changes in Growth, Fruit Quality, and Leaf Characteristics of Apple Tree (*Malus domestica* Borkh. ‘Fuji’) Grown under Elevated CO_2_ and Temperature Conditions. Hortic. Sci. Technol..

[34] Delgado-Baquerizo M., Guerra C.A., Cano-Díaz C., Egidi E., Wang J.-T., Eisenhauer N., Singh B.K., Maestre F.T. (2020). The proportion of soil-borne pathogens increases with warming at the global scale. Nat. Clim. Change.

[35] Balasooriya B.L.H.N., Dassanayake K., Ajlouni S. (2020). High temperature effects on strawberry fruit quality and antioxidant contents. Acta Hortic..

[36] Sikhandakasmita P., Kataoka I., Mochioka R., Beppu K. (2022). Impact of Temperatures During Fruit Development on Fruit Growth Rate and Qualities of ‘KU-PP2’ Peach. Hortic. J..

[37] Fischer G., Melgarejo L.M. (2021). Ecophysiological aspects of guava (*Psidium guajava* L.). A review. Rev. Colomb. De Cienc. Hortícolas.

[38] Chekanov K., Schastnaya E., Solovchenko A., Lobakova E. (2017). Effects of CO_2_ enrichment on primary photochemistry, growth and astaxanthin accumulation in the chlorophyte Haematococcus pluvialis. J. Photochem. Photobiol. B-Biol..

[39] Salto L., Maoz I., Goldenberg L., Carmi N., Porat R. (2024). Effects of Rainfall and Harvest Time on Postharvest Storage Performance of ‘Redson’ Fruit: A New Red Pomelo x Grapefruit Hybrid. Agriculture.

[40] Insausti P., Gorjón S. (2013). Floods affect physiological and growth variables of peach trees (*Prunus persica* (L.) Batsch), as well as the postharvest behavior of fruits. Sci. Hortic..

[41] Ye X., Li Y., Li X., Zhang Q. (2014). Factors influencing water level changes in China’s largest freshwater lake, Poyang Lake, in the past 50 years. Water Int..

[42] Slessarev E.W., Lin Y., Bingham N.L., Johnson J.E., Dai Y., Schimel J.P., Chadwick O.A. (2016). Water balance creates a threshold in soil pH at the global scale. Nature.

[43] Jat Baloch M.Y., Zhang W., Sultana T., Akram M., Shoumik B.A.A., Khan M.Z., Farooq M.A. (2023). Utilization of sewage sludge to manage saline–alkali soil and increase crop production: Is it safe or not?. Environ. Technol. Innov..

[44] Ihuoma S.O., Madramootoo C.A. (2017). Recent advances in crop water stress detection. Comput. Electron. Agric..

[45] Murcia-Asensi C., Fita A., de Luis-Margarit A., Guijarro-Real C., Raigon M.D., Blanca-Gimenez V., Diez-Diaz M., Rodriguez-Burruezo A. (2024). Grafting in Capsicum peppers as a strategy to mitigate the effects of climate change on yield and quality factors. Not. Bot. Horti Agrobot. Cluj-Napoca.

[46] Ferrarezi R.S., Qureshi J.A., Wright A.L., Ritenour M.A., Macan N.P.F. (2019). Citrus Production Under Screen as a Strategy to Protect Grapefruit Trees From Huanglongbing Disease. Front. Plant Sci..

[47] Hendricks G.S., Shukla S., Roka F.M., Sishodia R.P., Obreza T.A., Hochmuth G.J., Colee J. (2019). Economic and environmental consequences of overfertilization under extreme weather conditions. J. Soil Water Conserv..

[48] Copaciu F., Faur C.-A., Bunea A., Leopold L., Sima R.M., Lacatus M.A., Lupitu A., Moisa C., Copolovici D.M., Copolovici L. (2025). Enhancing UV-B Protection and Abiotic Stress Tolerance in Tomato Plants: The Role of Silicon Nanoparticles in Photosynthetic Parameters, Pigments, and Secondary Metabolite Production. Plants.

[49] Wang H., Guo Y., Zhu J., Yue K., Zhou K. (2021). Characteristics of Mango Leaf Photosynthetic Inhibition by Enhanced UV-B Radiation. Horticulturae.

[50] Bhattarai S., Jha D.K., Balyan S., Zhen S., Pillai S.S., Patil B.S. (2025). UV-B and blue light supplementation improves tomato quality and antioxidant dynamics: A novel approach for sustainable greenhouse production. J. Agric. Food Res..

[51] Sun M., Zhu Y., Jordan B., Wang T. (2024). Changes in Physiological Indices, Amino Acids, and Volatile Compounds in *Vitis vinifera* L. cv. Pinot Noir under UV-B Radiation and Water Deficit Conditions. Foods.

[52] Kim J.J., Fan R., Allison L.K., Andrew T.L. (2020). On-site identification of ozone damage in fruiting plants using vapor-deposited conducting polymer tattoos. Sci. Adv..

[53] Hui W., Hao X., Wang Y.-F., Heng Z., Tang M.-L., Du Y.-P. (2020). Ozone risk assessment of grapevine ‘Cabernet Sauvignon’ using open-top chambers. Sci. Hortic..

[54] Thwe A.A., Vercambre G., Gautier H., Pagès L., Jourdan C., Gay F., Kasemsap P. (2013). Dynamic shoot and root growth at different developmental stages of tomato (*Solanum lycopersicum* Mill.) under acute ozone stress. Sci. Hortic..

[55] Jian Z., Qi L., Xiaodong Z. (2023). Ocean acidification increases copper accumulation and exacerbates copper toxicity in Amphioctopus fangsiao (Mollusca: Cephalopoda): A potential threat to seafood safety. Sci. Total Environ..

[56] Doney S.C., Busch D.S., Cooley S.R., Kroeker K.J. (2020). The Impacts of Ocean Acidification on Marine Ecosystems and Reliant Human Communities. Annu. Rev. Environ. Resour..

[57] Yan W., Wang Z., Pei Y., Zhou B. (2024). Adaptive responses of eelgrass (*Zostera marina* L.) to ocean warming and acidification. Plant Physiol. Biochem..

[58] Delgado-Vargas V.A., Ayala-Garay O.J., Arevalo-Galarza M.d.L., Gautier H. (2023). Increased Temperature Affects Tomato Fruit Physicochemical Traits at Harvest Depending on Fruit Developmental Stage and Genotype. Horticulturae.

[59] Chuanhe L., Yan L. (2014). Effects of elevated temperature postharvest on color aspect, physiochemical characteristics, and aroma components of pineapple fruits. J. Food Sci..

[60] Du Toit A., Mpemba O., De Wit M., Venter S.L., Hugo A. (2021). The effect of size, cultivar and season on the edible qualities of nopalitos from South African cactus pear cultivars. S. Afr. J. Bot..

[61] Moretti C.L., Mattos L.M., Calbo A.G., Sargent S.A. (2010). Climate changes and potential impacts on postharvest quality of fruit and vegetable crops: A review. Food Res. Int..

[62] Fang L., Abdelhakim L.O.A., Hegelund J.N., Li S., Liu J., Peng X., Li X., Wei Z., Liu F. (2019). ABA-mediated regulation of leaf and root hydraulic conductance in tomato grown at elevated CO_2_ is associated with altered gene expression of aquaporins. Hortic. Res..

[63] Dong J., Xu Q., Gruda N., Chu W., Li X., Duan Z. (2018). Elevated and super-elevated CO_2_ differ in their interactive effects with nitrogen availability on fruit yield and quality of cucumber. J. Sci. Food Agric..

[64] Liu J., Hu T., Fang L., Peng X., Liu F. (2019). CO_2_ elevation modulates the response of leaf gas exchange to progressive soil drying in tomato plants. Agric. For. Meteorol..

[65] Modina D., Cola G., Bianchi D., Bolognini M., Mancini S., Foianini I., Cappelletti A., Failla O., Brancadoro L. (2023). Alpine Viticulture and Climate Change: Environmental Resources and Limitations for Grapevine Ripening in Valtellina, Italy. Plants.

[66] Kilic N., Burgut A., Gündesli M.A., Nogay G., Ercisli S., Kafkas N.E., Ekiert H., Elansary H.O., Szopa A. (2021). The Effect of Organic, Inorganic Fertilizers and Their Combinations on Fruit Quality Parameters in Strawberry. Horticulturae.

[67] Verma M.K. Climate change-Impact on Productivity and Quality of Temperate Fruits and Its Mitigation Strategies. Proceedings of the National workshop on Climate Change Impact, Mitigation, and Adaptation for Sustainable Horticulture.

[68] Gullino M.L., Albajes R., Al-Jboory I., Angelotti F., Chakraborty S., Garrett K.A., Hurley B.P., Juroszek P., Lopian R., Makkouk K. (2022). Climate Change and Pathways Used by Pests as Challenges to Plant Health in Agriculture and Forestry. Sustainability.

[69] Wang Y., Liu S., Wang H., Zhang Y., Li W., Liu J., Cheng Q., Sun L., Shen H. (2022). Identification of the Regulatory Genes of UV-B-Induced Anthocyanin Biosynthesis in Pepper Fruit. Int. J. Mol. Sci..

[70] Sun M., Jordan B., Creasy G., Zhu Y.-F. (2023). UV-B Radiation Induced the Changes in the Amount of Amino Acids, Phenolics and Aroma Compounds in *Vitis vinifera* cv. Pinot Noir Berry under Field Conditions. Foods.

[71] Zhu X., Trouth F., Yang T. (2023). Preharvest UV-B Treatment Improves Strawberry Quality and Extends Shelf Life. Horticulturae.

[72] Zardzewialy M., Matlok N., Piechowiak T., Balawejder M. (2023). The Influence of Ozonation Carried Out during Vegetation on the Content of Selected Bioactive Phytochemicals and the Microbiological Load of Tubers of *Raphanus sativus* var. *sativus*. Agriculture.

[73] Diaz-Lopez M., Galera L., Bastida F., Nicolas E. (2024). Tomato growth and physiology as well as soil physicochemical and biological properties affected by ozonated water in a saline agroecosystem. Sci. Total Environ..

[74] Li L., Yang B., Li J., Wang X., Ullah S. (2025). Effects of elevated atmospheric ozone concentration on biomass and non-structural carbohydrates allocation of cherry radish. Front. Plant Sci..

[75] Thor P., Perry D. (2025). Impact of climate change driven freshening, warming, and ocean acidification on the cellular metabolism of Atlantic Cod (*Gadus morhua*). Sci. Rep..

[76] Ullah I., Toor M.D., Yerlikaya B.A., Mohamed H.I., Yerlikaya S., Basit A., Rehman A.U. (2024). High-temperature stress in strawberry: Understanding physiological, biochemical and molecular responses. Planta.

[77] Lv Y., Fu A., Song X., Wang Y., Chen G., Jiang Y. (2023). 1-Methylcyclopropene and UV-C Treatment Effect on Storage Quality and Antioxidant Activity of ‘Xiaobai’ Apricot Fruit. Foods.

[78] Renforth P., Campbell J.S. (2021). The role of soils in the regulation of ocean acidification. Philos. Trans. R. Soc. B-Biol. Sci..

[79] Cheng Y.T., Zhang L., He S.Y. (2019). Plant-Microbe Interactions Facing Environmental Challenge. Cell Host Microbe.

[80] Singh B.K., Delgado-Baquerizo M., Egidi E., Guirado E., Leach J.E., Liu H., Trivedi P. (2023). Climate change impacts on plant pathogens, food security and paths forward. Nat. Rev. Microbiol..

[81] Ciliberti N., Fermaud M., Roudet J., Rossi V. (2015). Environmental Conditions Affect Botrytis cinerea Infection of Mature Grape Berries More Than the Strain or Transposon Genotype. Phytopathology.

[82] Nicolas Nguyen Van L., Vasseur V., Coroller L., Dantigny P., Le Panse S., Weill A., Mounier J., Rigalma K. (2017). Temperature, water activity and pH during conidia production affect the physiological state and germination time of *Penicillium* species. Int. J. Food Microbiol..

[83] Castro G., Perpiñá G., Esteras C., Armengol J., Picó B., Pérez-de-Castro A. (2020). Resistance in melon to Monosporascus cannonballus and M. eutypoides: Fungal pathogens associated with Monosporascus root rot and vine decline. Ann. Appl. Biol..

[84] War A.R., Taggar G.K., War M.Y., Hussain B. (2016). Impact of climate change on insect pests, plant chemical ecology, tritrophic interactions and food production. Int. J. Clin. Biol. Sci..

[85] Geng S., Hou H., Wang G., Jung C., Yin J., Qiao L. (2021). Temperature-dependent oviposition model of Scopula subpunctaria (Lepidoptera: Geometridae). J. Asia-Pac. Entomol..

[86] Kos J., Anic M., Radic B., Zadravec M., Hajnal E.J., Pleadin J. (2023). Climate Change-A Global Threat Resulting in Increasing Mycotoxin Occurrence. Foods.

[87] Eastburn D.M., Degennaro M.M., Delucia E.H., Dermody O., Mcelrone A.J. (2010). Elevated atmospheric carbon dioxide and ozone alter soybean diseases at SoyFACE. Glob. Change Biol..

[88] Khan M.R., Rizvi T.F. (2020). Effect of elevated levels of CO2 on powdery mildew development in five cucurbit species. Sci. Rep..

[89] Smet D., Depaepe T., Vandenbussche F., Callebert P., Nijs I., Ceulemans R., Van Der Straeten D. (2020). The involvement of the phytohormone ethylene in the adaptation of *Arabidopsis* rosettes to enhanced atmospheric carbon dioxide concentrations. Environ. Exp. Bot..

[90] Yucheng S., Huijuan G., Feng G. (2016). Plant–Aphid Interactions Under Elevated CO_2_: Some Cues from Aphid Feeding Behavior. Front. Plant Sci..

[91] Skendžić S., Zovko M., Pajač Živković I., Lešić V., Lemić D. (2021). Effect of Climate Change on Introduced and Native Agricultural Invasive Insect Pests in Europe. Insects.

[92] Romero F., Cazzato S., Walder F., Vogelgsang S., Bender S.F., van der Heijden M.G.A. (2022). Humidity and high temperature are important for predicting fungal disease outbreaks worldwide. New Phytol..

[93] Hull R. (2010). Effect of environmental conditions, and more particularly of soil moisture upon the emergence of peas. Ann. Appl. Biol..

[94] Cheng T., Tang J., Yang R., Xie Y., Chen L., Wang S. (2021). Methods to obtain thermal inactivation data for pathogen control in low-moisture foods. Trends Food Sci. Technol..

[95] Mamo B.E., Eriksen R.L., Adhikari N.D., Hayes R.J., Mou B., Simko I. (2021). Epidemiological Characterization of Lettuce Drop (*Sclerotinia* spp.) and Biophysical Features of the Host Identify Soft Stem as a Susceptibility Factor. PhytoFrontiers.

[96] Wakelin S.A., Gomez-Gallego M., Jones E., Smaill S., Lear G., Lambie S. (2018). Climate change induced drought impacts on plant diseases in New Zealand. Australas. Plant Pathol..

[97] Farigoule P., Chartois M., Mesmin X., Lambert M., Rossi J.-P., Rasplus J.-Y., Cruaud A. (2022). Vectors as Sentinels: Rising Temperatures Increase the Risk of Xylella fastidiosa Outbreaks. Biology.

[98] Lundin O., Rundlöf M., Jonsson M., Bommarco R., Williams N.M. (2021). Integrated pest and pollinator management–expanding the concept. Front. Ecol. Environ..

[99] Tossi V.E., Regalado J.J., Martinez J., Galvan A., Tosar L.J.M., Pitta-Alvarez S.I., Rebolloso M.M., Jamilena M. (2024). UV-B alleviates postharvest chilling injury of zucchini fruit associated with a reduction in oxidative stress. Postharvest Biol. Technol..

[100] Xiao Z., Wang J., Jiang N., Xiang X., Liu W. (2024). Metabolome and Transcriptome Analysis Provide Insights into Flower Bud Color Variation in the Adaptation to UV-B Radiation of Litchi. Agronomy.

[101] Fu S., Xue S., Chen J., Shang S., Xiao H., Zang Y., Tang X. (2021). Effects of Different Short-Term UV-B Radiation Intensities on Metabolic Characteristics of *Porphyra haitanensis*. Int. J. Mol. Sci..

[102] Migut D., Gorzelany J., Antos P., Balawejder M. (2019). Postharvest Ozone Treatment of Cucumber as a Method for Prolonging the Suitability of the Fruit for Processing. Ozone-Sci. Eng..

[103] Natalia M., Tomasz P., Malgorzata S., Maciej K., Pavel N., Ireneusz K., Maciej B. (2024). Modification of Fungicide Treatment Needs and Antioxidant Content as a Result of Real-Time Ozonation of Raspberry Plants. Molecules.

[104] Hashem M.Y., Ahmed S.S., Khalil S.S.H., El-Attar A.B., Abdelgawad K.F. (2024). Application of Modified Atmospheres to Control *Stegobium paniceum* and *Lasioderma serricorne* Infestation of Stored Chamomile and Coriander and Its Effect On Product Quality. J. Crop Health.

[105] Li L., Wang Y., Yu C., Li S., Lin T., Han S., Zhu T., Li S. (2023). Seasonal changes in the abundance *Fusarium proliferatium*, microbial endophytes and nutrient levels in the roots of hybrid bamboo *Bambusa pervariabilis* × *Dendrocalamopsis grandis*. Front. Plant Sci..

[106] Ukhurebor K., Aigbe U., Olayinka A., Nwankwo W., Emegha J. (2020). Climatic Change and Pesticides Usage: A Brief Review of Their Implicative Relationship. Assumpt. Univ.-Ejournal Interdiscip. Res. (AU-Ejir).

[107] van Munster M. (2020). Impact of Abiotic Stresses on Plant Virus Transmission by Aphids. Viruses.

[108] Zingales V., Taroncher M., Martino P.A., Ruiz M.-J., Caloni F. (2022). Climate Change and Effects on Molds and Mycotoxins. Toxins.

[109] Bencze S., Puskás K., Vida G., Karsai I., Balla K., Komáromi J., Veisz O. (2017). Rising atmospheric CO_2_ concentration may imply higher risk of Fusarium mycotoxin contamination of wheat grains. Mycotoxin Res..

[110] Vaughan M.M., Huffaker A., Schmelz E.A., Dafoe N.J., Christensen S., Sims J., Martins V.F., Swerbilow J., Romero M., Alborn H.T. (2014). Effects of elevated [CO_2_] on maize defence against mycotoxigenic usarium verticillioides. Plant Cell Environ..

[111] Pscheidt J.W., Heckert S. (2021). Progression of Kernel Mold on Hazelnut. Plant Dis..

[112] Aruna C., Das I.K., Reddy P.S., Ghorade R.B., Gulhane A.R., Kalpande V.V., Kajjidoni S.T., Hanamaratti N.G., Chattannavar S.N., Mehtre S. (2021). Development of Sorghum Genotypes for Improved Yield and Resistance to Grain Mold Using Population Breeding Approach. Front. Plant Sci..

[113] Bui T.A.T., Stridh H., Molin M. (2021). Influence of weather conditions on the quality of ‘Ingrid Marie’ apples and their susceptibility to grey mould infection. J. Agric. Food Res..

[114] Kang’ethe E.K., Gatwiri M., Sirma A.J., Ouko E.O., Mburugu-Musoti C.K., Kitala P.M., Nduhiu G.J., Nderitu J.G., Mungatu J.K., Hietaniemi V. (2017). Exposure of Kenyan population to aflatoxins in foods with special reference to Nandi and Makueni counties. Food Qual. Saf..

[115] Cui X., Ailijiang N., Mamitimin Y., Zhong N., Cheng W., Li N., Zhang Q., Pu M. (2023). Pollution levels, sources and risk assessment of polycyclic aromatic hydrocarbons in farmland soil and crops near Urumqi Industrial Park, Xinjiang, China. Stoch. Environ. Res. Risk Assess..

[116] Wang Z., Duan R., He Q., Liu H., Xu P., Wei M. (2025). Characteristics of airborne bacteria over inland and coastal atmosphere influenced by systemic air mass in northern China. Environ. Pollut..

[117] Bigirimana J., Uzayisenga B., Gut L.J. (2019). Population distribution and density of *Antestiopsis thunbergii* (Hemiptera: Pentatomidae) in the coffee growing regions of Rwanda in relation to climatic variables. Crop Prot..

[118] Chengkun Y., Xiaowen W., Wencan Z., Zhongrui W., Feili L., Hongxia W., Kaibing Z., Ake S., Minjie Q. (2024). Postharvest white light combined with different UV-B doses differently promotes anthocyanin accumulation and antioxidant capacity in mango peel. LWT-Food Sci. Technol..

[119] Santin M., Ranieri A., Hauser M.-T., Miras-Moreno B., Rocchetti G., Lucini L., Strid A., Castagna A. (2021). The outer influences the inner: Postharvest UV-B irradiation modulates peach flesh metabolome although shielded by the skin. Food Chem..

[120] Rangel K., Cabral F.O., Lechuga G.C., Carvalho J.P.R.S., Villas-Bôas M.H.S., Midlej V., De-Simone S.G. (2022). Detrimental Effect of Ozone on Pathogenic Bacteria. Microorganisms.

[121] Wang J., Zhang Y., Yu Y., Wu Z., Wang H. (2021). Combination of ozone and ultrasonic-assisted aerosolization sanitizer as a sanitizing process to disinfect fresh-cut lettuce. Ultrason. Sonochem..

[122] Goffi V., Magri A., Botondi R., Petriccione M. (2020). Response of antioxidant system to postharvest ozone treatment in ‘Soreli’ kiwifruit. J. Sci. Food Agric..

[123] Liu C., Chen C., Zhang Y., Jiang A., Hu W. (2021). Aqueous ozone treatment inhibited degradation of cellwall polysaccharides in fresh-cut apple during cold storage. Innov. Food Sci. Emerg. Technol..

[124] Lozowicka B., Jankowska M., Hrynko I., Kaczynski P. (2015). Removal of 16 pesticide residues from strawberries by washing with tap and ozone water, ultrasonic cleaning and boiling. Environ. Monit. Assess..

[125] Díaz-López M., Siles J.A., Ros C., Bastida F., Nicolás E. (2022). The effects of ozone treatments on the agro-physiological parameters of tomato plants and the soil microbial community. Sci. Total Environ..

[126] Brodowska A.J., Nowak A., Śmigielski K. (2018). Ozone in the food industry: Principles of ozone treatment, mechanisms of action, and applications: An overview. Crit. Rev. Food Sci. Nutr..

[127] Tiwari B.K., O’Donnell C.P., Patras A., Brunton N., Cullen P.J. (2009). Effect of ozone processing on anthocyanins and ascorbic acid degradation of strawberry juice. Food Chem..

[128] Gao W., Qu B., Yuan H., Song J., Li W. (2023). Heavy metal mobility in contaminated sediments under seawater acidification. Mar. Pollut. Bull..

[129] Umeoguaju F.U., Akaninwor J.O., Essien E.B., Amadi B.A., Igboekwe C.O., Ononamadu C.J., Ikimi C.G. (2023). Heavy metals contamination of seafood from the crude oil-impacted Niger Delta Region of Nigeria: A systematic review and meta-analysis. Toxicol. Rep..

[130] Su X., Yang X., Li H., Wang H., Wang Y., Xu J., Ding K., Zhu Y.-G. (2021). Bacterial communities are more sensitive to ocean acidification than fungal communities in estuarine sediments. Fems Microbiol. Ecol..

[131] Nan M., Xue H., Bi Y. (2022). Contamination, Detection and Control of Mycotoxins in Fruits and Vegetables. Toxins.

[132] Sun H., Sun Y., Jin M., Ripp S.A., Sayler G.S., Zhuang J. (2022). Domestic plant food loss and waste in the United States: Environmental footprints and mitigation strategies. Waste Manag..

[133] Ju K., He L., Li W., Ye Q., Zhou D., Wei X., Xu S. (2023). Put an eye on the future: Rethinking the theoretical price of fossil energy based on the perspective of intergenerational equity. Appl. Energy.

[134] Chu Q., Li H., Cannon N., Chang X., Feng J. (2025). An Evolutionary Game Analysis of Carbon Trading Mechanisms for Governments, Farmer Professional Cooperatives and Farmers. Systems.

[135] Gentil C., Basset-Mens C., Manteaux S., Mottes C., Maillard E., Biard Y., Fantke P. (2020). Coupling pesticide emission and toxicity characterization models for LCA: Application to open-field tomato production in Martinique. J. Clean. Prod..

[136] Yan M., Cheng K., Yue Q., Yan Y., Rees R.M., Pan G. (2016). Farm and product carbon footprints of China’s fruit production—Life cycle inventory of representative orchards of five major fruits. Environ. Sci. Pollut. Res..

[137] Chatterjee E. (2024). Towards an Energetics of Class: Comparing Energy Protests in India and the United States. Comp. Stud. Soc. Hist..

[138] Ymeri P., Gyuricza C., Fogarassy C. (2020). Farmers’ Attitudes Towards the Use of Biomass as Renewable Energy-A Case Study from Southeastern Europe. Sustainability.

[139] Alkaabneh F.M., Lee J., Gómez M.I., Gao H.O. (2021). A systems approach to carbon policy for fruit supply chains: Carbon tax, technology innovation, or land sparing?. Sci. Total Environ..

[140] Jia Z., Lin B., Liu X. (2023). Rethinking the equity and efficiency of carbon tax: A novel perspective. Appl. Energy.

[141] Saralegui-Diez P., Aguilera E., de Molina M.G., Guzman G.I. (2023). From field to table through the long way. Analyzing the global supply chain of Spanish tomato. Sustain. Prod. Consum..

[142] Hart M., Austin W., Acha S., Le Brun N., Markides C.N., Shah N. (2020). A roadmap investment strategy to reduce carbon intensive refrigerants in the food retail industry. J. Clean. Prod..

[143] Shen L., Lin F., Cheng T.C.E. (2022). Low-Carbon Transition Models of High Carbon Supply Chains under the Mixed Carbon Cap-and-Trade and Carbon Tax Policy in the Carbon Neutrality Era. Int. J. Environ. Res. Public Health.

[144] du Plessis M., van Eeden J., Goedhals-Gerber L. (2022). Carbon mapping frameworks for the distribution of fresh fruit: A systematic review. Glob. Food Secur..

[145] Zhang G., Xu J., Zhang Z., Chen W. (2024). Optimal decision-making and coordination of the shipping logistics service supply chain cooperation mode under the carbon quota and trading mechanism. Ocean Coast. Manag..

[146] Liu G., Hu J., Yang Y., Xia S., Lim M.K. (2020). Vehicle routing problem in cold Chain logistics: A joint distribution model with carbon trading mechanisms. Resour. Conserv. Recycl..

[147] Mesrzade P., Dehghanian F., Ghiami Y. (2023). A Bilevel Model for Carbon Pricing in a Green Supply Chain Considering Price and Carbon-Sensitive Demand. Sustainability.

[148] Malmgren E., Brynolf S., Styhre L., van der Holst J. (2023). Navigating unchartered waters: Overcoming barriers to low-emission fuels in Swedish maritime cargo transport. Energy Res. Soc. Sci..

[149] Moore N.a.d., Brehm J., Gruhl H. (2025). Driving innovation? Carbon tax effects in the Swedish transport sector. J. Public Econ..

[150] Walden P., Ollikainen M., Kahiluoto H. (2020). Carbon revenue in the profitability of agroforestry relative to monocultures. Agrofor. Syst..

[151] Staton T., Breeze T.D., Walters R.J., Smith J., Girling R.D. (2022). Productivity, biodiversity trade-offs, and farm income in an agroforestry versus an arable system. Ecol. Econ..

[152] Wang J., Wu W., Yang M., Gao Y., Shao J., Yang W., Ma G., Yu F., Yao N., Jiang H. (2024). Exploring the complex trade-offs and synergies of global ecosystem services. Environ. Sci. Ecotechnol..

[153] Edwards R., Halimatussadiah A., Moeis F.R., Maulia R.F. (2022). Agriculture, Development and Sustainability in the Covid-19 Era. Bull. Indones. Econ. Stud..

[154] Gren I.-M., Hoglind L., Jansson T. (2021). Refunding of a climate tax on food consumption in Sweden. Food Policy.

[155] Sebatjane M., Cardenas-Barron L.E., Nobil A.H. (2024). Sustainable inventory models fora three-echelon food supply chain with growing items and price- and carbon emissions-dependent demand under different emissions regulations. Clean. Logist. Supply Chain.

[156] Sager L. (2023). The global consumer incidence of carbon pricing: Evidence from trade. Energy Econ..

[157] Liu Z., Huang Y.-Q., Shang W.-L., Zhao Y.-J., Yang Z.-L., Zhao Z. (2022). Precooling energy and carbon emission reduction technology investment model in a fresh food cold chain based on a differential game. Appl. Energy.

[158] Yadav S., Khanna A. (2021). Sustainable Inventory Model for Perishable Products with Expiration Date and Price Reliant Demand Under Carbon Tax Policy. Process Integr. Optim. Sustain..

[159] Surya K.S., Fernandaz C.C., Karthikeyan C., Murugan P.P., Boomiraj K., Thamaraiselvi S.P., Manivasakan S., Rajashekar B. (2024). Constructing an innovative theoretical framework for tea grower’s adoption of carbon sequestration practices using PLS-SEM. Curr. Sci..

[160] Khan Z.A., Koondhar M.A., Khan A., Zhang Z., Ali U., Nurgazina Z., Liu T. (2023). Exploring the impact of carbon emissions and co-macroeconomic determinants on China’s sustainable apple export. Environ. Sci. Pollut. Res..

[161] Pu Z., He H., Ma C., Bo X., Yuan T., Chen P. (2024). The Impact of Consumer Utility on Duopoly Competition Markets Decision-Making Under Constraints of Carbon Cap Policies. Econ. Comput. Econ. Cybern. Stud. Res..

[162] Yang Y., Chen C. (2023). Analysis on the shadow price of carbon emissions from China’s forestry fruit industry-taking peaches as an example. Environ. Sci. Pollut. Res..

[163] Koiwanit J., Riensuwarn F., Palungpaiboon P., Pornchaloempong P. (2020). Business viability and carbon footprint of Thai-grown *Nam Dok Mai* mango powdered drink mix. J. Clean. Prod..

[164] Quiñónez Camarillo A.L., Schuhmann P.W., Randhir T., Orellana J. (2025). Coffee farmers willingness to accept payments for ecosystem services: Evidence from a choice experiment in Honduras. J. Environ. Manag..

[165] De Leijster V., Verburg R.W., Santos M.J., Wassen M.J., Martinez-Mena M., de Vente J., Verweij P.A. (2020). Almond farm profitability under agroecological management in southeastern Spain: Accounting for externalities and opportunity costs. Agric. Syst..

[166] Luo J., Huang M., Bai Y. (2024). Promoting green development of agriculture based on low-carbon policies and green preferences: An evolutionary game analysis. Environ. Dev. Sustain..

[167] Yan L., Wu Z., Liu J., Teo K.L. (2023). Pricing and Carbon Mitigation in a Dual-Channel Supply Chain: A Dynamic Game Approach. Emerg. Mark. Financ. Trade.

[168] Jiao Z.H., Duan H.W., Zhou Y.J., Xiang X.W. (2025). Low-carbon multimodal vehicle logistics route optimization with timetable limit using Particle Swarm Optimization. Adv. Prod. Eng. Manag..

[169] Dente S.M.R., Tavasszy L. (2018). Policy oriented emission factors for road freight transport. Transp. Res. Part D-Transp. Environ..

[170] Musavi M., Bozorgi-Amiri A. (2017). A multi-objective sustainable hub location-scheduling problem for perishable food supply chain. Comput. Ind. Eng..

[171] Stoessel F., Juraske R., Pfister S., Hellweg S. (2012). Life Cycle Inventory and Carbon and Water FoodPrint of Fruits and Vegetables: Application to a Swiss Retailer. Environ. Sci. Technol..

[172] Babagolzadeh M., Shrestha A., Abbasi B., Zhang Y., Woodhead A., Zhang A. (2020). Sustainable cold supply chain management under demand uncertainty and carbon tax regulation. Transp. Res. Part D-Transp. Environ..

[173] Wu R., Zhu L., Jiang M. (2024). Research on the evolution game of low-carbon operations in cold chain logistics considering environmental regulations and green credit. Heliyon.

[174] Sun J., Zhang X.-B., Liu Y., Zheng X. (2022). Pass-through of diesel taxes and the effect on carbon emissions: Evidence from China. J. Environ. Manag..

[175] Hussain J., Pan Y., Ali G., Yue X. (2020). Pricing behavior of monopoly market with the implementation of green technology decision under emission reduction subsidy policy. Sci. Total Environ..

[176] Zhong Z., Guo Z., Zhang J. (2021). Does the participation in global value chains promote interregional carbon emissions transferring via trade? Evidence from 39 major economies. Technol. Forecast. Soc. Change.

[177] Said Z., Vigneshwaran P., Shaik S., Rauf A., Ahmad Z. (2025). Climate and carbon policy pathways for sustainable food systems. Environ. Sustain. Indic..

[178] Torres P., Parvathikar S., Palys M.J. (2025). Techno-economic analysis of low-carbon ammonia and urea production in select African countries. Int. J. Hydrogen Energy.

[179] Steed C.A., Mercuur B.S., Mangaroo-Pilllay M. (2025). A sustainable decarbonisation roadmap for South African priority agro-processing sub-sectors. Energy Sustain. Dev..

[180] Palatnik R.R., Davidovitch A., Krey V., Sussman N., Riahi K., Gidden M. (2023). Accelerating emission reduction in Israel: Carbon pricing vs. policy standards. Energy Strategy Rev..

[181] He L., Han R., Hou L., Yue X. (2025). Product Line Design and Channel Configuration in Low-Carbon Supply Chains. IEEE Trans. Eng. Manag..

[182] Long Q., Wu Q., Jiang Y. (2024). On Short-term and Long-term Repeated Game Behavior- A Case Study of Oligopolistic Transportation Enterprises with Government Intervention under Fuzzy Environment. Appl. Artif. Intell..

[183] Alkahtani M., Khalid Q.S., Jalees M., Omair M., Hussain G., Pruncu C.I. (2021). E-Agricultural Supply Chain Management Coupled with Blockchain Effect and Cooperative Strategies. Sustainability.

[184] Clifton-Brown J., Hastings A., von Cossel M., Murphy-Bokern D., McCalmont J., Whittaker J., Alexopoulou E., Amaducci S., Andronic L., Ashman C. (2023). Perennial biomass cropping and use: Shaping the policy ecosystem in European countries. Glob. Change Biol. Bioenergy.

[185] Lim A.H., Holzer K. (2023). Trading in the era of carbon standards: How can trade, standard setting, and climate regimes cooperate?. Oxf. Rev. Econ. Policy.

[186] Varshney R.K., Bohra A., Yu J., Graner A., Zhang Q., Sorrells M.E. (2021). Designing Future Crops: Genomics-Assisted Breeding Comes of Age. Trends Plant Sci..

[187] Ahmar S., Hensel G., Gruszka D. (2023). CRISPR/Cas9-mediated genome editing techniques and new breeding strategies in cereals–current status, improvements, and perspectives. Biotechnol. Adv..

[188] Cirillo A., Graziani G., De Luca L., Cepparulo M., Ritieni A., Romano R., Di Vaio C. (2023). Minor Variety of Campania Olive Germplasm (“Racioppella”): Effects of Kaolin on Production and Bioactive Components of Drupes and Oil. Plants.

[189] Han A., Huang J., Wang X., Zhu Z. (2023). Efficient Water-Saving Irrigation, Space Efficiency and Agricultural Development—Study Based on Spatial Stochastic Frontier Model. J. Syst. Sci. Complex..

[190] Goh H.H., Xu Z., Liang X., Zhang D., Dai W., Liu H., Kurniawan T.A., Wong S.Y., Goh K.C. (2023). Solving carbon tax challenges with a holistic approach: Integrating evolutionary game theory and life cycle energy solutions. J. Clean. Prod..

[191] Shao Y., Zhou Z., Chen H., Zhang F., Cui Y., Zhou Z. (2022). The potential of urban family vertical farming: A pilot study of Shanghai. Sustain. Prod. Consum..

[192] Peng L., Yi J., Yang X., Xie J., Chen C. (2023). Development and characterization of mycelium bio-composites by utilization of different agricultural residual byproducts. J. Bioresour. Bioprod..

[193] Karimi Sani I., Masoudpour-Behabadi M., Alizadeh Sani M., Motalebinejad H., Juma A.S.M., Asdagh A., Eghbaljoo H., Khodaei S.M., Rhim J.-W., Mohammadi F. (2023). Value-added utilization of fruit and vegetable processing by-products for the manufacture of biodegradable food packaging films. Food Chem..

[194] Gao E., Cui Q., Jing H., Zhang Z., Zhang X. (2021). A review of application status and replacement progress of refrigerants in the Chinese cold chain industry. Int. J. Refrig..

[195] Liu P., Dong L., Cao A. (2020). Design of Medical Cold Chain Information Acquisition System Based on Linear Prediction. Wirel. Pers. Commun..

[196] Pan X., Guo S., Li M., Song J. (2021). The effect of technology infrastructure investment on technological innovation—A study based on spatial durbin model. Technovation.

[197] Wu Y., Liao Y., Xu M., He J., Tao Y., Zhou J., Chen W. (2022). Barriers identification, analysis and solutions to rural clean energy infrastructures development in China: Government perspective. Sustain. Cities Soc..

